# Stress-induced phase separation of ERES components into Sec bodies precedes ER exit inhibition in mammalian cells

**DOI:** 10.1242/jcs.260294

**Published:** 2022-12-01

**Authors:** Wessel van Leeuwen, Dan T. M. Nguyen, Rianne Grond, Tineke Veenendaal, Catherine Rabouille, Ginny G. Farías

**Affiliations:** ^1^ Hubrecht Institute of the KNAW & UMC Utrecht, Utrecht 3584 CT, The Netherlands; ^2^ Cell Biology, Neurobiology and Biophysics. Department of Biology, Faculty of Science, Utrecht University, Utrecht 3584 CH, The Netherlands; ^3^ Section Cell Biology, Center for Molecular Medicine, University Medical Center Utrecht, Utrecht 3584 CX, The Netherlands; ^4^ Department of Biomedical Sciences in Cells and Systems, UMC Groningen, Groningen 9713 AV, The Netherlands

**Keywords:** Sec body, Phase separation, Sec16, Stress, ER exit sites, ERES remodeling, Early secretory pathway, Mammalian cells, Protein transport

## Abstract

Phase separation of components of ER exit sites (ERES) into membraneless compartments, the Sec bodies, occurs in *Drosophila* cells upon exposure to specific cellular stressors, namely, salt stress and amino acid starvation, and their formation is linked to the early secretory pathway inhibition. Here, we show Sec bodies also form in secretory mammalian cells upon the same stress. These reversible and membraneless structures are positive for ERES components, including both Sec16A and Sec16B isoforms and COPII subunits. We find that Sec16A, but not Sec16B, is a driver for Sec body formation, and that the coalescence of ERES components into Sec bodies occurs by fusion. Finally, we show that the stress-induced coalescence of ERES components into Sec bodies precedes ER exit inhibition, leading to their progressive depletion from ERES that become non-functional. Stress relief causes an immediate dissolution of Sec bodies and the concomitant restoration of ER exit. We propose that the dynamic conversion between ERES and Sec body assembly, driven by Sec16A, regulates protein exit from the ER during stress and upon stress relief in mammalian cells, thus providing a conserved pro-survival mechanism in response to stress.

## Teaser

**Highlighted Article:** ER exit site (ERES) proteins undergo stress-induced redistribution into membraneless Sec bodies in mammals. This depletes them from the ER membrane, which impairs ER exit.

## INTRODUCTION

Phase separation is an important aspect of cellular organization. It is the result of demixing or coalescence of seemingly diffuse macromolecules into non-membrane-bound compartments. Phase separation occurs both in the cytoplasm and in the nucleus where it separates two distinct phases (Gomes and Shorter, 2019; Hyman et al., 2014). Interestingly, phase separation is often initiated and driven by ‘driver’ proteins that engage each other through low-affinity multivalent interactions (Banani et al., 2017). These driver proteins contain intrinsically disordered low-complexity domains that comprise repeating sequences and low amino acid variation (Franzmann and Alberti, 2019; Martin and Mittag, 2018). Absence of driver proteins leads to unstable phase separated compartments or they will not form.

Phase separation can also be driven by stress (such as oxidative stress, ER stress, heat shock and nutrient starvation), resulting in the formation of stress assemblies (van Leeuwen and Rabouille, 2019). One of the most-studied stress assemblies are P-bodies and stress granules that form around RNAs (Jain and Parker, 2013; Wheeler et al., 2016). Previously, a new stress-driven phase separated compartment has been identified in *Drosophila* S2 cells, namely the Sec body (Zacharogianni et al., 2014).

Sec bodies are related to the early secretory pathway, a major anabolic pathway used by 30% of proteins to reach their functional localizations (Sharpe et al., 2010). They form at the endoplasmic reticulum (ER) exit sites (ERES), ribosome-free regions of the ER where newly synthesized proteins destined to nearly all membrane compartments of the cell, as well as to the extracellular space, exit via COPII-coated vesicles. In S2 cells, Sec bodies contain COPII subunits that form the COPII coat itself, the inner coat proteins Sec23 and Sec24 and the outer coat proteins Sec13 and Sec31 (Miller and Schekman, 2013). They also contain the ERES large peripheral scaffold protein Sec16, a key regulator in the organization of the ERES and in COPII-coated vesicle budding (Sprangers and Rabouille, 2015).

In *Drosophila* S2 cells, Sec bodies form upon specific stresses that drive these ERES components (Sec16, Sec23 and Sec31) to coalescence into micrometer-sized membraneless assemblies. Sec bodies form as one to five rounded structures per cell (Zacharogianni et al., 2014; Zhang et al., 2021), and their diameter ranges from 0.4 to 1 μm. They are membrane-less, polyadenylated RNA-free and display liquid-like properties (Zacharogianni et al., 2014). Sec bodies are reversible upon stress removal, and they contribute to cell survival (Aguilera-Gomez et al., 2016; Zacharogianni et al., 2014). In addition, Sec16 has been shown to be a driver for Sec body formation in S2 cells. Its depletion prevents Sec body formation, and overexpression of a conserved 44-amino-acid C-terminal domain of Sec16, serum responsive domain conserved (SRDC), drives Sec body formation, even in the absence of stress (Aguilera-Gomez et al., 2016).

Although Sec bodies are well characterized in *Drosophila* cells, their formation has remained unknown in mammalian cells. Here, we show that in the insulin-producing cell line INS1, NaCl stress and amino acid starvation leads to the formation of Sec bodies that are positive for Sec16A, Sec16B and COPII subunits. These structures are membraneless and liquid droplet-like, with the ability to fuse. We show that Sec16A, but not Sec16B, is a driver for Sec body formation. Finally, we show that Sec body formation precedes the inhibition of ER exit and likely is a cause of this inhibition. Our findings suggest that dynamic assembly and disassembly of Sec bodies regulates protein secretion in mammalian cells during periods of stress and stress relief.

## RESULTS

### Upon NaCl stress and KRBm incubation, Sec16A is redistributed into large structures resembling Sec bodies

Recent work in *Drosophila* S2 cells has shown that Sec body formation is promoted by two specific types of stress. First, by the addition of a high concentration of NaCl to the growing medium, and second, by incubating the cells in Krebs Ringer bicarbonate buffer (KRB) leading to a moderate NaCl stress that is potentiated by the absence of amino acids (Zacharogianni et al., 2014; Zhang et al., 2021).

**Fig. 1.**
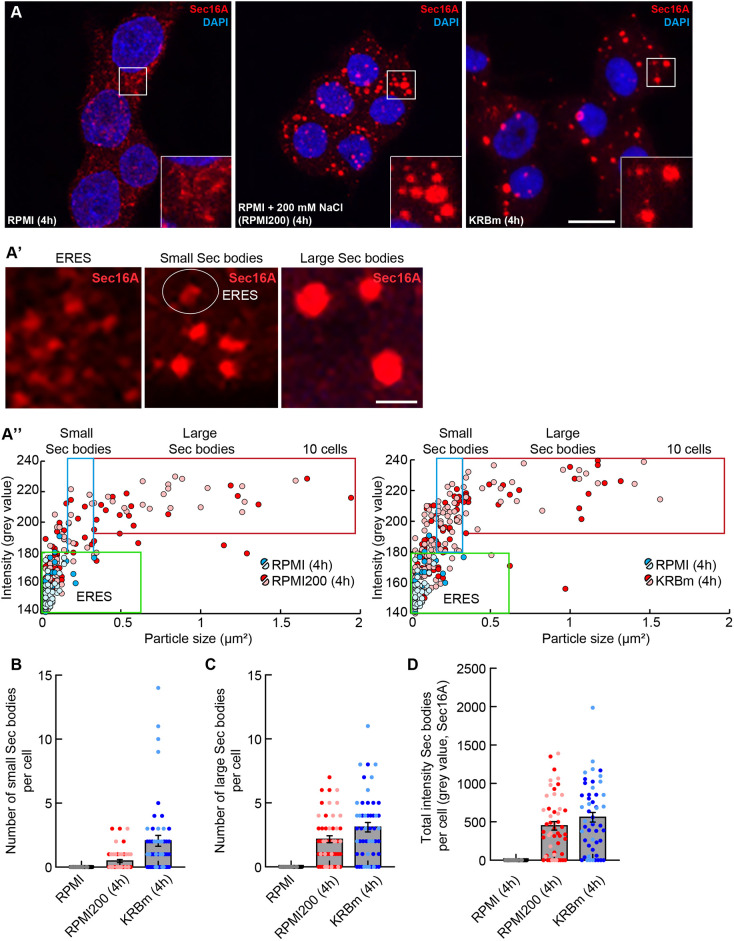
**Coalescence of ERES into structures resembling Sec bodies in INS-1 cells upon salt stress and KRBm.** (A) IF visualization of endogenous Sec16A (red) in INS-1 cells upon incubation in RPMI, RPMI200 (RPMI+200 mM NaCl) and KRBm (4 h). Note that, upon stress, Sec16A redistributes into small and large round structures that resemble S2 cells Sec bodies. (A′) Representative images of ERES in control cells (left), and small Sec bodies and large Sec bodies in cells upon stress. The white circle highlights the presence of a remaining ERES upon stress. (A″) Scatterplot depicting Sec16A foci size and intensity upon incubation in RPMI, RPMI200 and KRBm (4 h). All foci in the red box are determined as ‘large Sec bodies’, foci in the blue box are ‘small Sec bodies’ and foci in the green box are ERES. The values within the red and blue box were used to determine the total intensity of Sec bodies per cell and per condition. Foci from a total of 10 cells are displayed. (B,C) Dot plot depicting the number of small (B) and large (C) Sec16A-positive Sec bodies per cell in INS-1 cells upon RPMI, RPMI200 and KRBm (4 h) culture. (D) Dot and bar plot depicting the total intensity of Sec16A-positive Sec bodies per cell in INS-1 cells upon RPMI, RPMI200 and KRBm (4 h). *N*=2 experiments, *n*=50 cells in B–D. See also Figs S1 and S2. Scale bars: 10 μm (A); 1 μm (A′). Error bars are s.e.m. (B–D).

To assess whether Sec bodies also form in mammalian cells, we first incubated a range of mammalian cell lines, including HepG2, MDCKII and MRC5, with addition of 250 mM NaCl for 4 h to their growing medium. This led to some redistribution of the ERES protein Sec16A, but this was not as pronounced as in S2 cells and occurred only in a low percentage of cells (Fig. S1A). We then assessed rat insulinoma INS-1 cells, a highly secretory cell type that secretes insulin (Cline et al., 2011). Incubation of INS-1 cells in RPMI medium supplemented with 200 mM NaCl (RPMI200) led to a remarkable coalescence of endogenous Sec16A into large structures. In basal conditions, Sec16A localizes at ERES (Hughes et al., 2009; Tillmann et al., 2015; Watson et al., 2006), but upon high NaCl, it coalescences into round and bright enlarged structures that resemble S2 cell Sec bodies (Fig. 1A,A′).

**Fig. 2.**
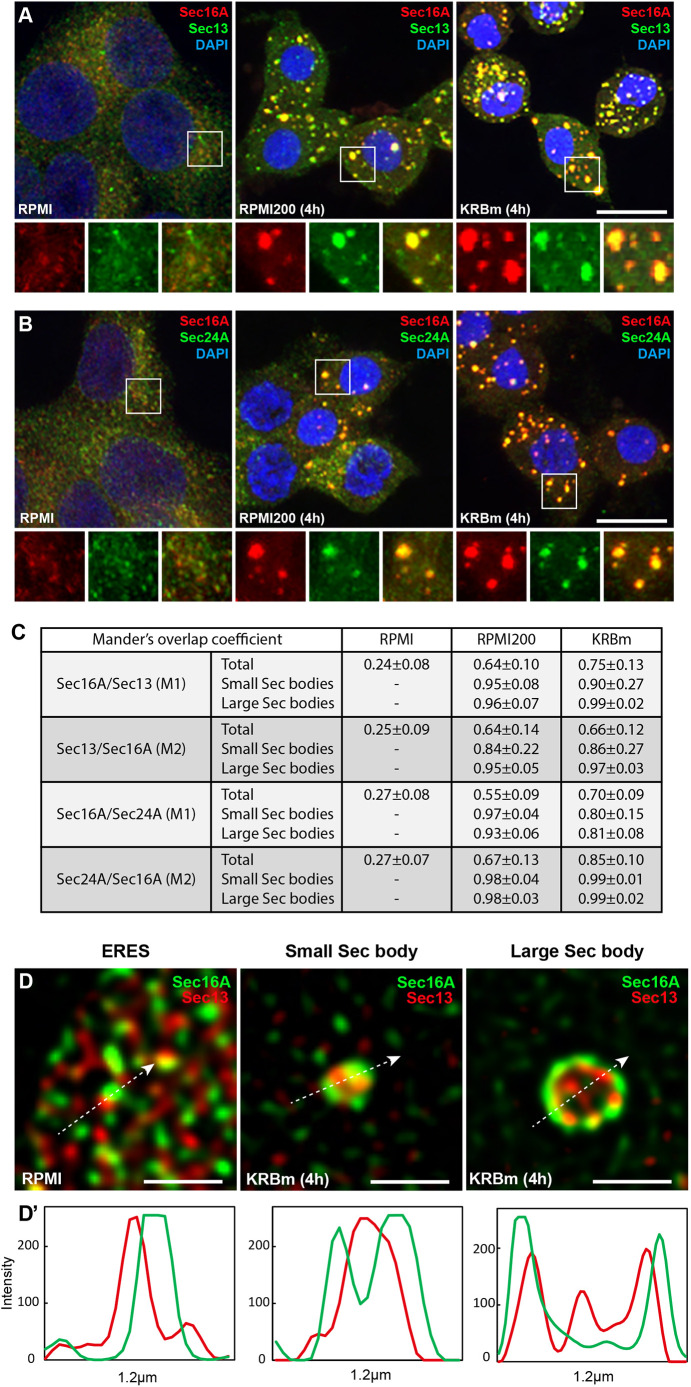
**Sec16A-positive structures contain COPII subunits.** (A,B) IF visualization of endogenous Sec16A and Sec13A (A), and Sec16A and Sec24A (B) in INS-1 cells upon incubation in RPMI, RPMI200 and KRBm (4 h). (C) Quantification of the Mander's overlap coefficient (M1 and M2) for experiments in A and B. Values correspond to the total overlap of proteins per cell, or the overlap per small or large Sec body. *N*=2 experiments, *n*=12–37 cells, 32–86 structures per condition, and are given as mean±s.d. (D,D′) Representative super-resolution STED images of ERES, and small and large Sec bodies from cells incubated in KRBm and labeled as in A. Intensity profile line for the arrow shown in D (D′). Notice the shell-like distribution of Sec16A around the COPII protein Sec13 within Sec bodies. See also Fig. S3. Scale bars: 10 μm (A,B), 1 μm (D).

**Fig. 3.**
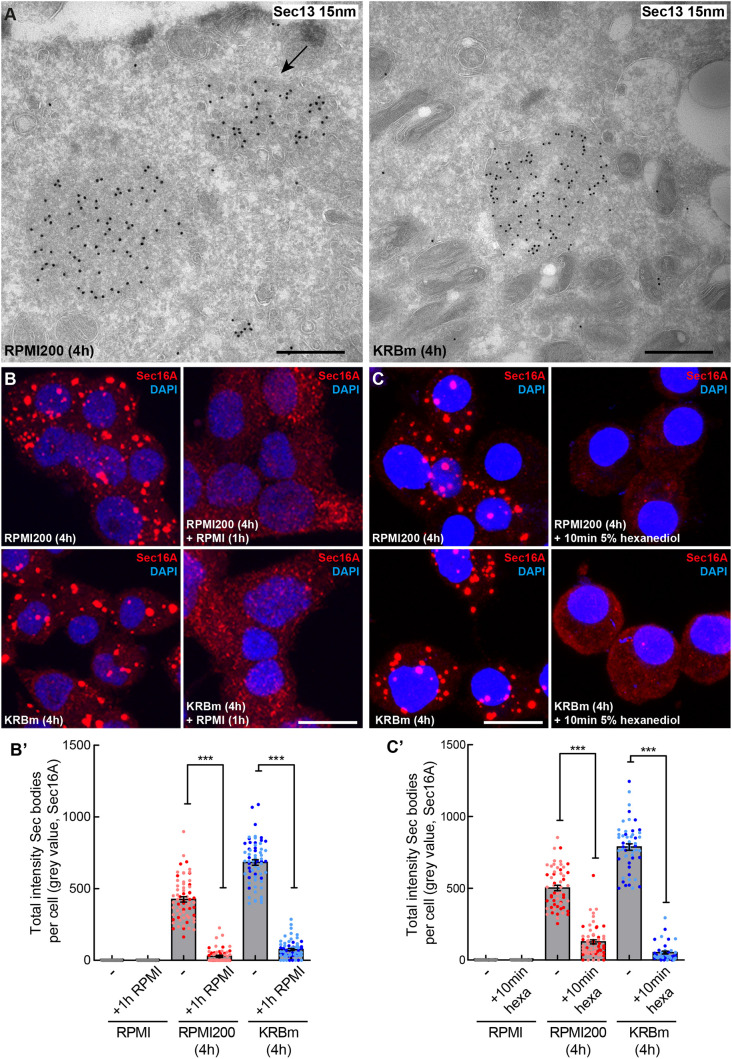
**Sec16A-positive structures in stressed INS-1 cells are Sec bodies.** (A,A′) Visualization of endogenous Sec13 (15 nm PAG) by immunoelectron microscopy in ultrathin frozen sections of INS-1 cells incubated in RPMI200 and KRBm for 4 h. Sec13 is concentrated in structures (Sec bodies) that are slightly denser than the surrounding cytoplasm, are round, are not sealed in a lipid bilayer and are in close proximity to the ER. Arrow points to a remaining ERES that have not remodeled upon stress. (B,B′) IF visualization of Sec16A in INS-1 cells after 4 h in RPMI200 and KRBm followed by 1 h in RPMI (B). Note that Sec body dissolution is complete within 1 h of stress removal and that Sec16A is localized again at ERES. Quantification in B′; *N*=2 experiments, *n*=64–69 cells. (C,C′) IF visualization of Sec16A in INS-1 cells treated with 5% hexanediol (hexa) for 10 min after incubation in RPMI200 or KRBm for 4 h (C). Quantification in C′; *N*=2 experiments, *n*=40–60 cells. Scale bars: 500 nm (A), 10 μm (B) and (C). Error bars are s.e.m. (B′,C′). ***P<0.001 (Mann–Whitney test).

We then designed a method to assess the efficiency of Sec16A redistribution in INS-1 cells considering the number, size (area) and mean intensity of coalesced structures, and whether they were small and large (Fig. 1A′,A″). For the sake of simplicity, we will name these structures Sec bodies, as we show below that they are (see Figs 2 and 3). ERES are defined as irregular small structures (100–150 structures) with low fluorescence intensity after immunofluorescence (IF) staining of Sec16A (Fig. 1A′,A″, green box). Small Sec bodies are brighter and slightly larger structures of 0.15–0.3 μm^2^ (Fig. 1A′,A″, red box), and large Sec bodies are round, bright and have a size of >0.3 μm^2^ (Fig. 1A′,A″, blue box; see also Materials and Methods).

Accordingly, we found that RPMI200 leads to the formation of an average of 0.5 small and 2.2 large Sec bodies per cell (Fig. 1A′–C).

By multiplying the size and mean intensity of each small and large structure, the Sec16A redistribution can also be represented as the total intensity of all Sec bodies. This gives a clear read-out of Sec body formation upon stress (Fig. 1D). Upon addition of NaCl, Sec16A redistributes into large structures in a time-dependent manner, in which 4 h triggers a more efficient coalescence, when compared to that of shorter incubation periods (Fig. S1B). They are present in 72% of the incubated cells (Fig. 1D).

As in S2 cells, the formation of these structures in INS-1 cells is specific for addition of NaCl, as addition of 200 mM either sodium acetate or KCl to RPMI does not lead to their formation. Neither does the addition of 0.4 M sorbitol, indicating that osmotic shock is not involved in this response (Fig. S1C,C′).

In parallel, incubation of INS-1 cells in KRB buffer for mammalian cells (KRBm, which induces a moderate NaCl stress combined with amino acid starvation) also leads to the efficient formation of Sec bodies (Fig. 1A,A′) in a time-dependent manner (Fig. S1B). After 4 h of KRBm incubation, cells displayed an average of 2.1 small and 3.1 large Sec bodies per cell, similar to what was seen with high NaCl (Fig. 1B–D), in 78% of the incubated cells (Fig. 1D).

Overall, this data indicates that, as in S2 cells, both NaCl stress and amino acid starvation in KRBm leads to the redistribution of ERES of INS-1 cells into structures that morphologically resemble Sec bodies. Importantly, primary culture of rat neurons incubated in high NaCl (NB125) and KRBm also display similar structures, showing that Sec body formation is a general mechanism in response to stress at least in secretory mammalian cells (Fig. S2A,A′).

### Stress-induced Sec16A-positive structures are Sec bodies

To determine whether the enlarged Sec16A-positive coalesced structures observed in INS-1 cells are indeed Sec bodies, we first assessed whether they also contain COPII subunits, a documented feature of *Drosophila* Sec bodies. Accordingly, we found that endogenous Sec13 and Sec24A colocalize with Sec16A under both RPMI200 and KRBm (Fig. 2A,B), with high Mander's overlap coefficient for both stress conditions (M1 and M2 values in Fig. 2C). Importantly, both small and large Sec bodies contain COPII components, displaying similar values for Mander's overlap (Fig. 2C). Similar co-distribution of ERES proteins within Sec bodies was observed in neurons (Fig. S2B,B′).

We further studied the co-distribution of Sec bodies by STED super-resolution microscopy, and confirmed that Sec16A and Sec13 were both present in Sec bodies (small and large) (Fig. 2D). We noticed that in large Sec bodies, Sec16 appeared to form an outer layer, or ‘shell’, surrounding Sec13 (Fig. 2D). This sub-compartmentalization was also observed in small Sec bodies, where Sec16A molecules started remodeling around Sec13 (Fig. 2D).

In addition, we investigated whether these stress-induced structures contain other proteins of the early secretory pathway. p115 (also known as USO1), a protein that facilitates COPII vesicle transport from the ER to the Golgi apparatus (Sapperstein et al., 1995; Waters et al., 1992), partially colocalized with Sec16A-positive Sec bodies (Fig. S3A), but not GM130, GRASP65 and GRASP55 (also known as GOLGA2, GORASP1 and GORASP2, respectively) (Fig. S3B–D). Overall, Sec bodies in INS-1 cells do not appear to incorporate a set of peripheral Golgi proteins.

A second critical feature of Sec bodies in S2 cells is that they are not enclosed by a sealed lipid membrane (Zacharogianni et al., 2014). To unravel this feature in stressed INS-1 cells, we employed immunoelectron microscopy (IEM) to visualize their morphology after labeling with an antibody against endogenous Sec13. Either upon RPMI200 or KRBm, Sec13-positive coalescences appeared as slightly electron-dense structures with a diameter between 0.3 and 1 μm. Consistent with being a ‘membrane-less’ assembly, the Sec13-positive structures were not surrounded by membrane, but were often positioned in close proximity to the ER membrane (Fig. 3A).

Furthermore, consistent with formation by phase separation, we found that these structures were largely dissolved within 1 h of stress relief (i.e. further incubation in RPMI), and that Sec16A had regained its typical ERES pattern (Fig. 3B,B′).

Finally, phase separation can lead to liquid as well as solid assemblies, both of which are reversible (Saad et al., 2017; Wheeler et al., 2016). To assess this feature for the Sec16A-positive structures triggered by stress in INS-1 cells, we used 1,6-hexanediol, an aliphatic alcohol that has been used to differentiate between the two states of membrane-less assemblies (Kroschwald et al., 2015; Peskett et al., 2018). Liquid assemblies are sensitive to this hexanediol and dissolve, whereas solid assemblies do not. Addition of 5% hexanediol for 10 min to RPMI200 and KRBm-stressed INS-1 cells led to the complete dissolution of the Sec16A-positive structures (Fig. 3C,C′), suggesting that they are liquid-like assemblies. Of note, 1,6-hexanediol also dissolves the typical ERES in non-stressed cells, suggesting that ERES themselves are also liquid-like assemblies (Gallo et al., 2020 preprint). Overall, these data indicate that INS-1 redistributed Sec16A-positive structures have liquid-like features.

Taken together, these results indicate that high salt stress and amino acid starvation in KRBm leads to the formation of bona fide Sec bodies. They contain COPII subunits, and they are membrane-less reversible stress assemblies with features of liquid droplets.

### Sec16A is a driver in Sec body formation

As mentioned in the Introduction, phase separation is often driven by proteins that contain disordered regions. The sequence of the single *Drosophila* Sec16 homolog comprises mostly disordered regions (except for the central conserved domain). We have shown that Sec16 is a driver for Sec body formation in *Drosophila* S2 cells; its depletion prevents Sec body formation and the overexpression of the 44-amino-acid peptide, namely SRDC, located in the C-terminus of the protein is enough to drive Sec body formation (Aguilera-Gomez et al., 2016).

**Fig. 4.**
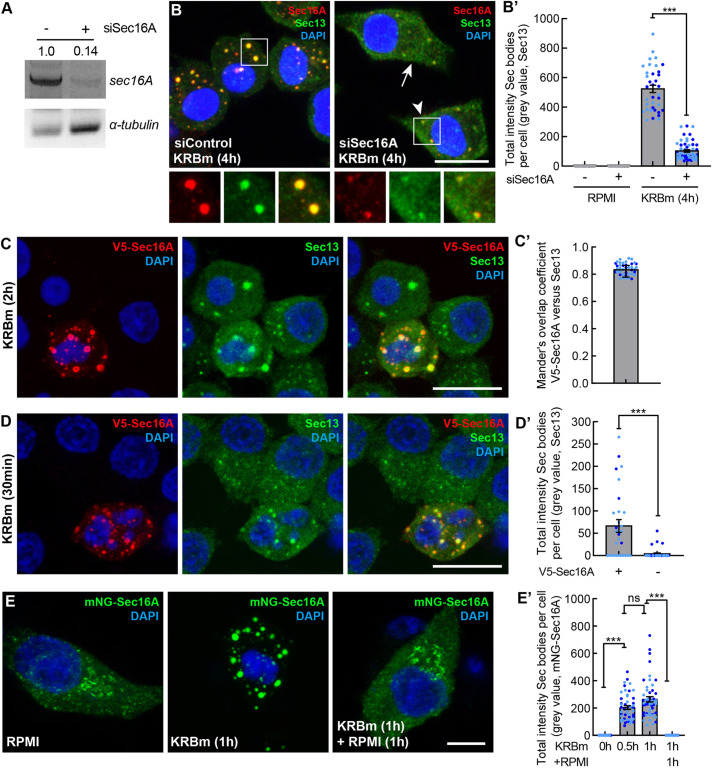
**Sec16A drives Sec body formation in INS-1 cells.** (A) PCR of Sec16A and α-tubulin cDNAs from mock and Sec16A-depleted (siSec16A) INS-1 cells. Note that depletion of Sec16A for 4 days leads to a very efficient knockdown (86%). Normalized value of Sec16A against α-tubulin, was used to calculate the Sec16A ratio between mock and Sec16A-depleted cells. Results shown are representative for two repeats. (B,B′) IF visualization of Sec16A and Sec13 in Sec16A-depleted INS-1 cells incubated in KRBm for 4 h (B). Arrow, cell with undetectable Sec16A; arrowhead, cell with a small pool of Sec16A. Note that Sec body formation (marked by Sec13) is inhibited upon Sec16A depletion. Quantification of the total intensity of Sec bodies per cell (B′); *N*=2 experiments, *n*=36–70 cells. (C,C′) IF visualization of V5–Sec16A (anti V5 and endogenous Sec13 in V5-Sec16A transfected INS-1 cell after KRBm incubation for 2 h (C). Mander's overlap coefficient between V5 and Sec13 in Sec bodies (C′); *N*=2 experiments, *n*=40 cells. (D,D′) IF visualization of V5–Sec16A (anti V5) and endogenous Sec13 in V5–Sec16A transfected INS-1 cell after KRBm incubation for 30 min (D). Compare the Sec body formation in transfected versus adjacent non-transfected cells. Quantification of total Sec13-positive Sec body intensity per cell in transfected and non-transfected cells (D′); *N*=2 experiments, *n*=29–36 cells. (E,E′) Fluorescence images of INS-1 cells expressing mNeonGreen (mNG)–Sec16A incubated in KRBm for 0, 60 min, and 60 min followed by 1 h further incubation in RPMI (E). Quantification of mNG–Sec16A total intensity of Sec bodies (E′); *N*=2 experiments, *n*= 50–51 cells. See also Fig. S4. Scale bars: 10 μm (B–D); 5 μm (E). Error bar are s.d. (C′), s.e.m. (B′,D′,E′). ***P<0.001; ns, not significant (Mann–Whitney test (B′,D′); Kruskal-Wallis test followed by Dunn′s multiple comparisons (E′)).

Rat Sec16A also harbors a large amount of disordered region in its sequence and contains a conserved 44-residue SRDC (Fig. S4A,B) that is instrumental in the formation of Sec bodies in *Drosophila* cells (Aguilera-Gomez et al., 2016). In this respect, we tested whether it is also a driver in Sec body formation in INS-1 cells. Knockdown of Sec16A using siRNA (that specifically depletes *Sec16A* mRNA by 86%; Fig. 4A) led to a strong inhibition of Sec body formation (visualized with endogenous Sec13), both upon KRBm incubation (Fig. 4B,B′) and RPMI200 (Fig. S4C,C′). The inhibition of Sec body formation was stronger in the cells where Sec16A was hardly detectable by IF (arrow in Fig. 4B). In cells that still exhibited a small pool of Sec16A, the resulting Sec13-positive structures were much smaller than typical Sec bodies (arrowhead in Fig. 4B). These data demonstrate that Sec16A is essential for the formation of Sec bodies in INS-1 cells.

As Sec16A appears to be a driver in Sec body formation, we then asked whether its overexpression is also able to induce Sec body formation. Upon overexpression of V5-tagged Sec16A, we observed its localization in Sec bodies upon KRBm incubation for 2 h (Fig. 4C). The formation of these structures was stress specific, as tagged V5–Sec16A localized to ERES in cells growing in RPMI (Fig. S4D). Importantly, V5–Sec16A colocalized with endogenous Sec13 (Fig. 4C), with a Mander's coefficient of ∼0.8 (Fig. 4C′) showing that V5-tagged Sec16A is incorporated into Sec bodies upon KRBm incubation for 2 h as endogenous Sec16A is.

We then used a shorter KRBm incubation (30 min). The reasoning is that in non-transfected INS-1 cells Sec body formation is only observed in 11% of the cells (Fig. 4D,D′, non-transfected), consistent with the low efficiency of short KRBm incubation to induce Sec body formation (Fig. S1B). If Sec16A were a driver, Sec bodies might form more efficiently upon transfection. Indeed, V5–Sec16A overexpression led to Sec13-positive Sec body formation in 62% of the cells, a 6-fold increase (Fig. 4D,D′). These results confirm Sec16A as a driver for Sec body formation.

To further verify this, we visualized Sec body formation using mNeonGreen (mNG) fluorescently tagged Sec16A. Again, Sec body formation occurred quickly in cells expressing mNG–Sec16A upon a 30- and 60-min incubation in KRBm (Fig. 4E,E′). Importantly, incubation of cells with RPMI for 1 h after 1 h KRB treatment caused Sec body disassembly, leading to a re-localization of mNG–Sec16A similar to what was seen in control cells (Fig. 4E,E′). This result indicates that tagged Sec16A induces formation of reversible Sec bodies.

### Sec16B is also a component of Sec bodies but is not a driver

Mammalian genomes contain two distinct genes encoding for Sec16 proteins (Sec16A and Sec16B) (Bhattacharyya and Glick, 2007). Both proteins localize to ERES in mammalian cells. Unlike Sec16A, Sec16B does not contain the SRDC in its C-terminus or anywhere else in its sequence, but it does display a similar extent of disordered regions, especially in the C-terminal region (Fig. S4E). Interestingly, these two proteins have overlapping functions, yet they do not compensate for each other (Budnik et al., 2011). When either are depleted, ER exit is inhibited (Bhattacharyya and Glick, 2007).

**Fig. 5.**
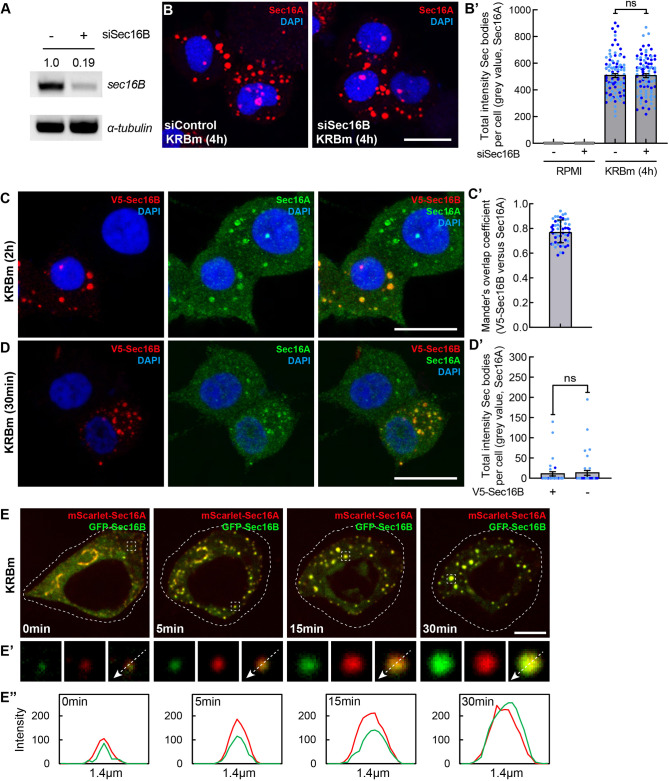
**Sec16B is a component of Sec bodies but does not drive their formation.** (A) PCR of Sec16B and α-tubulin cDNAs from mock and Sec16B-depleted INS-1 cells. Note that depleting Sec16B for 4 days leads to a very efficient knockdown (81%). Normalized value of Sec16B against α-tubulin, was used to calculate the Sec16B ratio between mock and Sec16B-depleted cells. Results shown are representative for two repeats. (B,B′) IF visualization of Sec16A in Sec16B-depleted INS-1 cells incubated in KRBm for 4 h (B). Note that Sec body formation (marked by Sec16A) is not inhibited upon Sec16B depletion. Quantification of the total intensity of Sec bodies per cell (B′); *N*=2 experiments, *n*=81–99 cells. (C,C′) IF visualization of V5-Sec16B (anti V5) and endogenous Sec16A in V5-Sec16A transfected INS-1 cell after KRBm incubation for 2 h (C). Mander's overlap coefficient of V5 and Sec16A in Sec bodies (C′); *N*=2 experiments, *n*=51 cells. (D,D′) IF visualization of V5–Sec16B (anti V5) and endogenous Sec16A in V5–Sec16B transfected INS-1 cells after KRBm incubation 30 min. Quantification of Sec16A-positive Sec body total intensity per cell in transfected and non-transfected cells in D′; *N*=2 experiments, *n*=35 and 42 cells. (E–E″) Representative still images of time-points 0, 5, 15 and 30 min from a live cell expressing mScarlet-Sec16A and GFP-Sec16B and treated with KRBm during live cell imaging (E). Single and merged channels of selected ERES and Sec body structures (E′). The cell outline is highlighted by a dashed line. Intensity profile lines displaying co-distribution of Sec16A and Sec16B for the arrow shown in E′ (E″). See also Movie 2. See also Fig. S4. Scale bars: 10 μm (B–D); 5 μm (E). Error bars are s.d. (C′), s.e.m. (B′,D′). ns, not significant (Mann–Whitney test).

To assess the role of Sec16B in Sec body formation, we specifically depleted it using specific siRNAs, leading to an 81% reduction in the *Sec16B* mRNA level (Fig. 5A). However, in stark contrast with Sec16A depletion, Sec16B depletion did not prevent Sec body formation neither upon KRBm incubation (Fig. 5B,B′) nor upon NaCl stress (Fig. S4F,F′).

This might result from the fact that Sec16B is not a Sec body component. To test this, we overexpressed V5-tagged Sec16B, which in control conditions localized to ERES, as expected (Fig. S4G). Similar to Sec16A, Sec16B was efficiently incorporated into Sec bodies upon KRBm incubation for 2 h (Fig. 5C) where it co-localized with endogenous Sec16A (Fig. 5C), with a Mander's coefficient of ∼0.77 (Fig. 5C′). These results show that Sec16B is also a component of Sec bodies. However, unlike Sec16A (Fig. 4D,D′), overexpression of V5-Sec16B did not promote Sec body formation after KRBm incubation for 30 min (Fig. 5D,D′). This suggests that Sec16B is a component of Sec bodies but not a driver of their formation.

Given this differential role in driving Sec body formation, we asked whether Sec16A and Sec16B are recruited to the same forming Sec bodies. To address this, we expressed fluorescently tagged mScarlet–Sec16A and GFP–Sec16B and visualized transfected cells by live-cell imaging upon KRBm incubation. Critically, each Sec body contained both Sec16 species that perfectly colocalized and were recruited to Sec bodies with the same kinetics (Fig. 5E–E″).

Taken together, these results strongly indicate that both Sec16A and Sec16B are components of Sec bodies, but that only Sec16A is a driver for stress-induced Sec body formation in mammalian cells.

### ERES components are simultaneously recruited into Sec bodies that form by fusion

We then questioned the mechanism behind the formation of Sec bodies in INS-1 cells. INS-1 cell Sec bodies are sensitive to hexanediol, suggesting that they are liquid droplets. One of the properties of liquid droplets is their propensity to fuse with one another, especially small ones fusing with larger ones, the so-called ‘droplet fusion’ (or coarsening) effect (Brangwynne et al., 2009; Weber and Brangwynne, 2012), leading to coalescence of a given size.

**Fig. 6.**
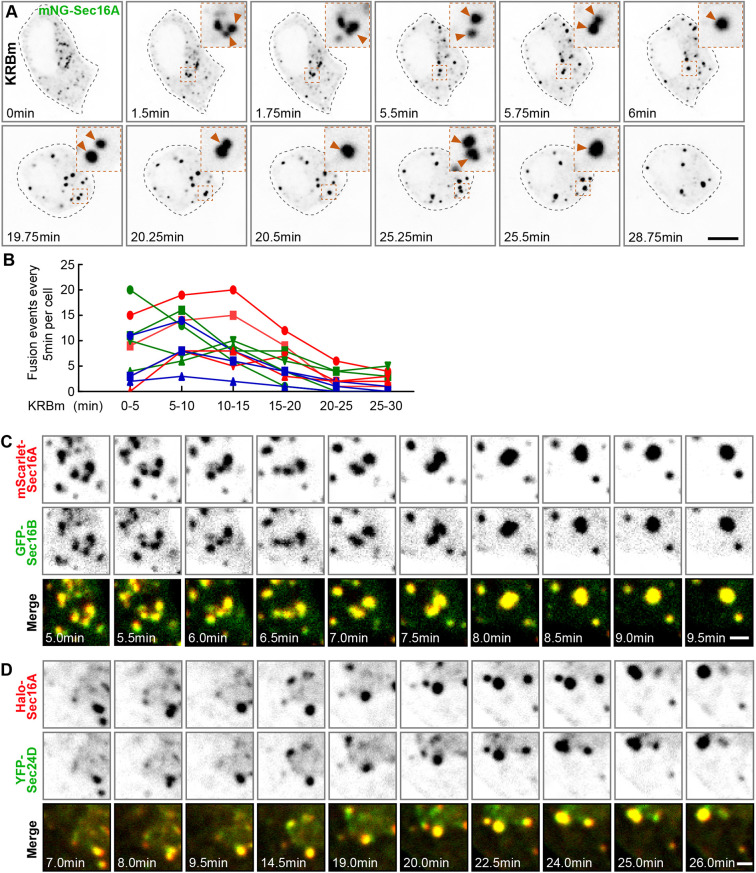
**Sec body assembly is mediated by fusion of small foci containing several ERES components.** (A) Representative still images of a live INS-1 cell transfected with mNeonGreen (mNG)–Sec16A and recorded every 15 s for 30 min during KRBm incubation. Arrowheads in inserts despite fusion events. The cell outline is highlighted by a dashed line. See also Movie 1. (B) Quantification of number of fusion events occurring during 5 min for 30 min KRBm incubation. In the graph, lines correspond to individual cells undergoing fusion upon KRBm treatment; *N*=2 experiments, *n*=11 cells. (C) Representative still images of a region of a live INS-1 cell expressing mScarlet–Sec16A and GFP–Sec16B imaged every 30 s for 30 min during KRBm incubation. Note that mScarlet–Sec16A and GFP–Sec16B are simultaneously recruited into newly formed Sec bodies. See also Fig. 5E and Movie 2. (D) Representative still images of a region of an INS-1 cell expressing Halo-Sec16A and YFP-Sec24D. Cells were pre-incubated with the permeable Halo-646 dye prior to imaging. Live-imaged as in C. Simultaneous recruitment of Halo-Sec16A and YFP-Sec24D into assembling Sec bodies is shown. See also Fig. S5 and Movie 3. Sequences in C,D are representative of 12–17 cells examined. Scale bars: 5 μm (A), 1 μm (C,D).

To study the dynamics of Sec body formation, we performed live-cell imaging of mNG–Sec16A every 15 s for 30 min during KRBm treatment. Live-cell imaging revealed that ERES started reorganizing into small round foci within a few minutes of KRBm treatment. These foci then fused with each other over time, leading to the formation of typical Sec bodies within 30 min of treatment (Fig. 6A; Movie 1). We quantified the total number of fusion events occurring during 5 min, in intervals, until the 30 min endpoint. Interestingly, we observed that the number of fusion events peaked at 10–15 min. After 20 min, fusion events were still observed but were fewer as newly formed Sec bodies reached a stable size of 0.5–1 μm in diameter (Fig. 6B; Movie 1).

We have shown above that Sec16A and Sec16B colocalize in large Sec bodies (Fig. 5C,E–E″)*.* We have also shown that Sec bodies contain COPII subunits (Fig. 2). Using live-cell imaging, we then asked whether these different ERES components are either sequentially or simultaneously recruited into forming Sec bodies. We observed that mScarlet–Sec16A and GFP–Sec16B co-distributed in most of the small foci that were observed just after the beginning of the KRBm treatment (Fig. 5E–E″, Fig. 6C; Movie 2). These dually positive small foci underwent further fusion events until Sec bodies reached their large typical size (Fig. 6C, Movie 2). Similar results were observed in cells expressing Halo–Sec16A and YFP–Sec24D (Fig. 6D; Fig. S5, Movie 3).

These results indicate that Sec bodies form by fusion of remodeled ERES, and that the different ERES components are recruited simultaneously into newly formed Sec bodies.

### Testing the relationship between Sec body formation and ER exit

Finally, we addressed the role of stress on the inhibition of the early secretory pathway. Indeed, extracellular stress is known to influence protein transport (Farhan and Rabouille, 2011; van Leeuwen et al., 2018). More specifically, given that Sec bodies contain both Sec16 orthologs and most of the COPII subunits, we assessed the role of Sec bodies with regards to the inhibition of protein transport out of the ER. In *Drosophila* cells, the formation of Sec bodies in stressed cells correlates with transport inhibition in the secretory pathway (Zacharogianni et al., 2014). However, the question as to whether Sec body formation causes inhibition of protein transport (and ER exit in particular), remains to be answered.

**Fig. 7.**
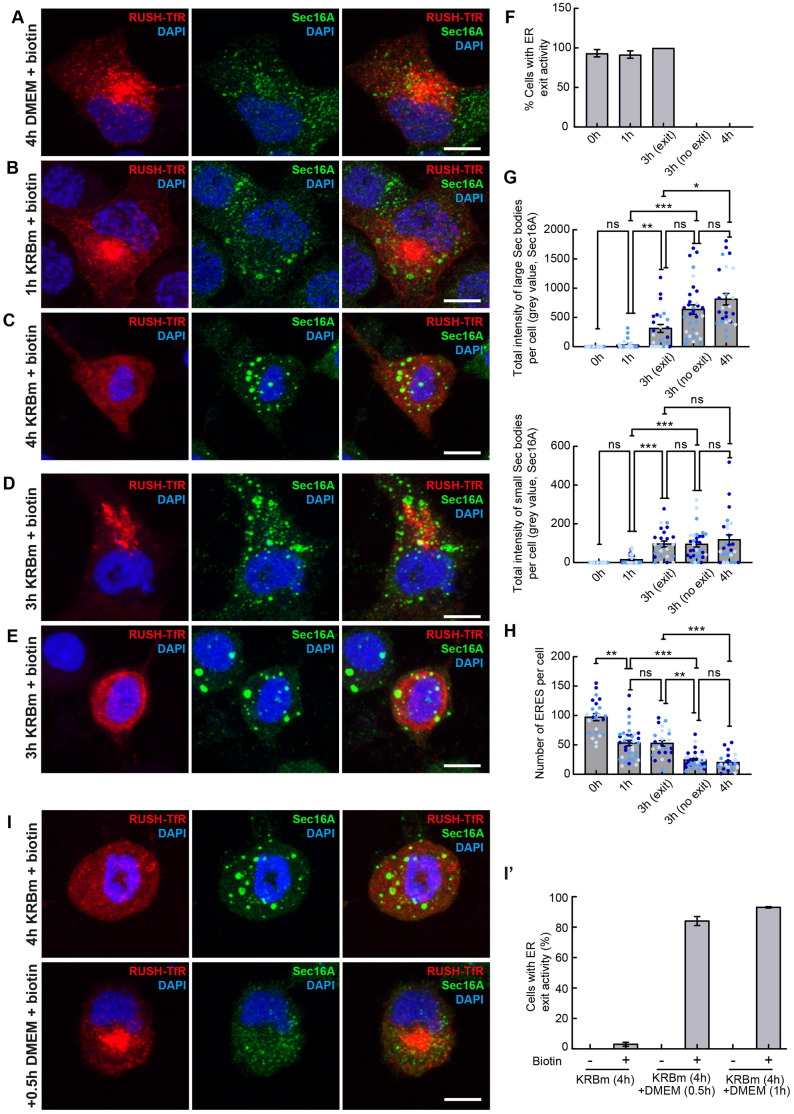
**Sec body formation precedes ER exit.** (A–C) Visualization of RUSH-TfR and endogenous Sec16A upon addition of biotin (30 min) in INS-1 cells cultured in DMEM (4 h) (A), KRBm (1 h) (B) and KRBm (4 h) (C). (D,E) Visualization of RUSH-TfR and endogenous Sec16A upon addition of biotin (30 min) in cells in KRBm for 3 h. The release of RUSH-TfR out of the ER is observed in 43% of the cells (D), whereas RUSH-TfR is retained in the ER in the remaining 57% of the cells (E). (F–H) Quantification of ER exit activity (F), total intensity of large and small Sec bodies per cell (G), and number of ERES per cell (H) in cells treated as indicated in A–E. ER exit activity is 100% and 0% for the two groups at 3 h KRBm, as we selected cells by this parameter for subsequent quantification. *N*=3 experiments, *n*=24–36 cells. (I,I′) Visualization of RUSH-TfR and endogenous Sec16A in cells incubated in KRBm for 4 h followed by 30 min (0.5 h) or 1 h in DMEM (including 30 min biotin, 100 μM). Note that the Sec bodies have dissolved and that RUSH-TfR has efficiently exited the ER. Quantification of the ER exit (I′); *N*=2 experiments, *n*=27–31 cells. See also Figs S6, S7 and Table S1*.* Scale bars: 10 μm (A–E,I). Error bars are s.e.m. (F–H,I′); *P<0.05; **P<0.01; ***P<0.001; ns, not significant (Kruskal–Wallis test followed by Dunn's multiple comparisons).

To test whether Sec body formation in INS-1 cells also coincides or correlates with inhibition of trafficking in the early secretory pathway, we used the RUSH system to study the ER exit of the transferrin receptor (TfR) (Boncompain et al., 2012). As expected, Strep-KDEL-Halo-Transferrin Receptor (TfR)-SBP (RUSH-TfR) is retained in the ER when cells are incubated in DMEM without biotin (Fig. S6A). The addition of biotin for 30 min led to the release of RUSH-TfR out of the ER in 93% of the cells (Fig. 7A,F) and reached a compartment largely positive for giantin (also known as GOLGB1) (Fig. S6B). Under this condition, biotin-induced cargo release from the ER did not cause remodeling of ERES (Fig. S6D).

We then asked what happens to protein exit from the ER in cells upon amino acid starvation in KRBm. To assess this, we incubated transfected cells for increasing times in KRBm and then added biotin for the last 30 min of this incubation. If ER exit is inhibited upon stress, RUSH-TfR will be retained in the ER, even after the addition of biotin. If the ER exit is still active, it will exit the ER.

After 1 h KRBm (including 30 min biotin), RUSH-TfR exited the ER and reached the Golgi in 92% of the cells, showing that ER exit is still active (Fig. 7B,F). In the absence of biotin, RUSH-TfR was retained in the ER as expected (Fig. S6C). In this population of cells, hardly any Sec bodies (small or large) have formed (Fig. 7B,G; Table S1). This shows that the mere incubation in KRBm is not sufficient to impede ER exit. In contrast, after 4 h incubation in KRBm (including 30 min biotin), RUSH-TfR was retained in the ER in 100% of the cells, showing that ER exit is fully inhibited (Fig. 7C,F). These cells exhibit the full complement of Sec bodies (Fig. 7C,G; Table S1). Therefore, these results show a strong correlation between the formation of Sec bodies and inhibition of ER exit. Furthermore, as we have not observed a colocalization of RUSH-TfR with Sec bodies, these results also demonstrate that Sec bodies are transport incompetent.

We then asked whether ER exit inhibition is a contributing factor for Sec body formation. The effect of Sec16A and Sec16B depletion suggests that ER exit inhibition is not a driving factor, as both depletions lead to this inhibition (Bhattacharyya and Glick, 2007), but Sec bodies do not form upon either depletion in non-stressed cells. To confirm that the sole inhibition of ER exit does not lead to Sec body formation, non-stressed INS-1 cells were treated with Brefeldin A (BFA; which inhibits trafficking in the early secretory pathway, including in 99% of INS-1 cells; Fig. S7A,A′) and H89 [which leads to the translocation of Sar1 away from the ER membrane (Lee and Linstedt, 2000), and causes ER exit inhibition in 53% of INS-1 cells, Fig. S7B,B′]. Neither, BFA- nor H89-treated cells that displayed disrupted ER exit, showed any coalescence of ERES components into Sec bodies (Fig. S7).

To test whether inhibition of ER exit is a contributing factor to Sec body formation, BFA was added to cells incubated in KRBm. This treatment did not affect Sec body formation (Fig. S7C,C′). This shows that the inhibition of the ER exit is not a contributing factor in Sec body formation.

### Sec body formation controls ER exit by regulating the number of functional ERES

The above results suggest that ER exit inhibition could be a consequence of Sec body formation. To test this, we examined the cells after 3 h incubation in KRBm (including 30 min biotin). There, two populations of cells were observed that responded differentially. In the first (57% of the cells), RUSH-TfR was retained in the ER and Sec body formed as efficiently as in 4 h of KRBm, both in term of number, sizes and intensities for small and large Sec bodies (Fig. 7E–G; Table S1). In the second population (43% of the cells), RUSH-TfR had exited the ER. However, the formation of Sec bodies was clearly visible, although more moderately than in the first population, for both for small and large Sec bodies (Fig. 7D,F,G; Table S1). Taken together, these results shows that Sec body formation precedes ER exit inhibition.

To understand what distinguishes these two populations of cells, those where ER exit occurs from those where it does not, we quantified the number of ERES in cells of each population. In cells treated with KRBm for 3 h where RUSH-TfR exited the ER, the number of ERES was half of what it is in DMEM (53 versus 97/cell, respectively; Fig. 7H) a number (53) that is comparable to that in the cells incubated in KRBm for 1 h (54), where ER exit is very efficient (Fig. 7H). The decrease in the ERES number from 97 to 54 upon 1 h KRBm, is likely due to their initial reorganization into slightly larger and brighter structures (Fig. S6E) and might correspond to the first fusion events observed by live-cell imaging (Fig. 6A). In contrast, in cells where ER exit does not occur, the number of ERES had dropped to 27 (Fig. 7H), a number very similar to that in cells incubated in KRBm for 4 h (23) where ER exit is fully blocked (Fig. 7H).

These results show that Sec body formation precedes the inhibition of ER exit, and that Sec body formation is not a consequence of ER exit inhibition. Instead, we propose that cells are competent for ER exit until the number of ERES has dropped by 75% of what it is in DMEM. This suggests that Sec body formation recruits or titrates away Sec16A (and the other COPII subunits) from ERES, leading to their progressive depletion from ERES that then become non-functional. In other words, ER exit is inhibited when Sec16A and COPII subunits are incorporated substantially into Sec bodies and no longer available at ERES.

To test this further, we monitored RUSH-TfR transport upon stress relief (i.e. in cells incubated in KRBm for 4 h followed by either 30 min or 1 h back in DMEM). This condition led to the complete dissolution of Sec bodies and the re-localization of Sec16A to ERES (Figs 3B, 7I). Strikingly, this was accompanied by the exit of RUSH-TfR from the ER in 90% of the cells upon biotin addition (Fig. 7I,I′) whereas it was properly retained in the ER when biotin is not added (Fig. S6F).

Taken together, this experiment shows that, in INS-1 cells, Sec bodies act as a storage for critical ERES components, such as Sec16A, controlling its availability to ERES functioning in ER exit. It also shows that ERES components are quickly available again upon stress relief, leading to active protein transport.

## DISCUSSION

### Sec bodies are conserved in evolution – a key stress response?

Remodeling of ERES into membraneless Sec bodies has been observed in *Drosophila* S2 cells upon stress (Zhang et al., 2021). In this study, we show that Sec bodies also form in mammalian cells (INS-1 cells and primary neurons) upon same stress-specific conditions. Mammalian Sec bodies share several characteristics with *Drosophila* Sec bodies. They recruit Sec16 protein(s) and COPII subunits, and are membranelles and reversible structures. In mammalian cells, the two Sec16 orthologs, Sec16A and Sec16B, are both recruited into Sec bodies but only Sec16A is a Sec body driver. The redistribution of ERES proteins into Sec bodies occurs by means of fusion events in which ERES components are simultaneously recruited into forming Sec bodies.

In contrast to stress granules, which form upon different types of stress (heat shock, ER stress, sodium arsenite and osmotic stress) (Aulas et al., 2018; van Leeuwen and Rabouille, 2019), studies in *Drosophila* cells have shown that Sec bodies form only upon specific types of stress, namely, high NaCl stress and upon amino acid starvation in KRB (Zhang et al., 2021). This is also true for the mammalian cells we used in this study, which reveals a strong convergent evolution, potentially underlying a fundamental ancient stress response. Interestingly, Sec16 and all COPII subunits are conserved in last eukaryotic common ancestor, suggesting that they could also respond to stress in ancient cellular lineages (Schlacht and Dacks, 2015).

Although the ERES reorganization into Sec bodies is very prominent in INS-1 and primary neurons, it is also observed in many other mammalian cell lines (especially what appears to be small Sec bodies), although at lower efficiency. Both INS-1 cells and neurons have an active secretion, and this suggests that secretory cells could have more efficient mechanisms to protect the secretory pathway upon stress. The abundance of the Sec body driver Sec16A, and the signaling behind their formation might also play a role in the efficiency of Sec body formation.

### Sec body form by fusion in close proximity to the ER

One important feature of membraneless organelles is that they are not enclosed by a lipid membrane. Sec bodies are not delimited by membrane, but they are in close proximity to the ER membrane. This interaction with the ER is consistent with the first step of their biogenesis through the fusion of ERES. This suggests that the ERES components that form Sec bodies do not disperse prior to their coalescence but rather coalesce *in situ*. The reorganized small structures (larger ERES) continue to fuse with each other to form small and then large Sec bodies, until they reach an optimal and stable size ∼1 μm in diameter. Another characteristic of membraneless organelles is that they are reversible. We observed that Sec bodies efficiently disassemble and restore ERES, even after just 30 min of stress removal. This fast and efficient response to stress relief might be related to the close contact with the ER membrane. It remains elusive how Sec bodies disassemble after stress relief, either through dispersion of ERES components, perhaps by active signaling as for stress granules (Wippich et al., 2013), or through fission events. Supporting the latter model, recent findings have identified a role of the ER in controlling membraneless P-bodies size by promoting fission events (Lee et al., 2020). It is likely that the close contact of Sec bodies with the ER facilitates a fast restoration of ERES on ER membrane.

### Sec bodies do not contain Golgi proteins

We have observed different ERES components recruited into INS-1 Sec bodies, such as Sec16A, Sec16B and the COPII subunits Sec13 and Sec24D (and likely Sec31 as in *Drosophila* S2 cells; Zacharogianni et al., 2014). Here, we show that ERES components are simultaneously recruited to forming Sec bodies, in agreement with the initial coalescence of functional ERES containing all required components. Conversely, Golgi proteins are largely excluded from Sec bodies, except for a small amount of p115. p115 is associated with the ER-to-Golgi transport machinery and has been shown to regulate ERES (Alvarez et al., 1999, 2001; Kondylis and Rabouille, 2003; Sapperstein et al., 1995). It is therefore likely that the ERES-located p115 is incorporated into Sec bodies. This is also consistent with a recent proteomic analysis of Sec bodies in *Drosophila* S2 cells, where p115 is also found as part of the Sec body proteome (our unpublished results). Interestingly, GRASP55 and GRASP65 appear to form larger structures upon stress but those are not Sec bodies (marked by Sec16A). Whether those are related to compartments for unconventional protein secretion (CUPS), which also form upon nutrient starvation, remains to be elucidated (Cruz-Garcia et al., 2014). This suggests that nutrient stress leads to an important remodeling of the cytoplasm that somehow remains distinct and sustains different functions.

### Sec body formation is driven by Sec16A

Phase separation is often promoted by driver proteins. These proteins induce phase separation through a mild change in their conformation. This leads to their coalescence, which then recruits other proteins through a low-affinity interaction (Banani et al., 2017). The absence of drivers prevents phase separation (Banani et al., 2017, 2016).

Here, we show that although the two mammalian Sec16 orthologs are present in INS-1 cells, Sec16A but not Sec16B is a driver of Sec body formation, in agreement with *Drosophila* Sec16 also being a driver (Aguilera-Gomez et al., 2016). Mammalian Sec16A shares a high similarity with *Drosophila* Sec16 (dSec16) (Ivan et al., 2008). As for dSec16, Sec16A contains low complexity intrinsically disordered sequences that could potentially stimulate Sec body formation by engaging low-affinity multivalent interactions with other ERES proteins. However, Sec16B also contains such disordered sequences, yet it is not a driver. This suggests that the presence of disordered sequences in Sec16A and Sec16B are likely necessary for their incorporation into Sec bodies, but not sufficient for driving their coalescence.

Interestingly, the STED microscopy revealed that Sec16A forms a shell surrounding a core of Sec13 and possibly other COPII subunits. This sub-compartmentalization is reminiscent of what is seen with *Caenorhabditis elegans* P granules, where the intrinsically disordered protein MEG-3 forms a shell around a core of PLG-3 (Putnam et al., 2019). This sub-compartmentalization is also in line with that observed for different membraneless assemblies, including P-bodies of the *Drosophila* oocytes (Weil et al., 2012), and stress granules (van Leeuwen and Rabouille, 2019).

### Sec bodies and ER exit

Finally, we propose that the remodeling of ERES into Sec bodies causes a depletion of functional ERES, and thus they participate in shutting down the early secretory pathway during stress, which is efficiently restored after stress relief. Given the relationship between stress assemblies and the modulation of anabolic pathways, it is likely that Sec body formation is a key conserved mechanism of cell survival during stress, and fitness upon stress relief (Kroschwald and Alberti, 2017; van Leeuwen and Rabouille, 2019), through their capacity of modulating protein secretion.

We find that Sec body formation precedes inhibition of ER exit and protein secretion in the secretory pathway, showing that their formation is not the consequence of this ER inhibition. In other words, ER exit persists even when cells have already formed Sec bodies, as long as there are enough functional ERES. Instead, it appears that ER exit is inhibited when the number of functional ERES drops below a threshold and when their components (such as Sec16A and COPII subunits) are quantitatively recruited to Sec bodies. Sec16A is critical in COPII dynamics (Bhattacharyya and Glick, 2007; Hughes et al., 2009; Joo et al., 2016; Tillmann et al., 2015; Wilhelmi et al., 2016). Without Sec16A and COPII components at ERES, protein transport is slowed down, and cells proliferation is compromised (Tillmann et al., 2015). It is possible that the titrating away of key components, making cells less competent for ER exit, is accompanied by stress-induced mechanisms independent of Sec body formation. These could be modifications of, and other effects of the stress, on other parts of the machinery that are independent from the ERES remodeling we report here.

### Model – Sec bodies, ER exit and survival

Why do Sec bodies form? From studies in *Drosophila* S2 cells, we have previously proposed that protein recruitment into Sec bodies protects them from stress-mediated degradation in such a way that they are available upon stress relief. Here, we show that Sec body formation might participate in shutting down ER exit. As secretion is an energy-consuming process (Brandizzi and Barlowe, 2013; Depaoli et al., 2019; Südhof and Rothman, 2009; Yong et al., 2019), the inhibition of its first step through Sec body formation would save energy that could be redirected to other processes important to cope with the stress itself. This is in line with the general notion that cellular stress leads to a slowing down of secretion (Farhan and Rabouille, 2011; Tillmann et al., 2013).

Sec bodies might simply store key ERES components in a near-native state, allowing them to be quickly functional upon stress relief. This reinforces the notion that Sec bodies are pro-survival (as shown in *Drosophila* cells) in line with the general concept that the formation of stress assemblies is a key cellular mechanism to cope with stress and allow cells to thrive upon stress relief (Kroschwald and Alberti, 2017; Riback et al., 2017).

## MATERIALS AND METHODS

### Cell culture and treatments

INS-1 cells and INS-1 823/3 cells (Sigma-Aldrich, scc208) were cultured in RPMI-1640 medium, GlutaMAX Supplement, HEPES (Gibco, 72400047), 10% FBS (Sigma-Aldrich, F7524), 1% penicillin/streptomycin (Gibco, 15140122) and 50 μM 2-mercaptoethanol (Sigma-Aldrich, M6250). Cells were maintained at 37°C and 5% CO_2_.

Sec bodies were formed in INS-1 cells by incubating cells with either RPMI-1640 medium plus 200 mM NaCl (RPMI200) or Krebs Ringer Bicarbonate buffer modified (KRBm) for 4 h. KRBm contains 0.7 mM Na_2_HPO_4_, 1.5 mM NaH_2_PO_4_, 30 mM NaHCO_3_, 237 mM NaCl, 4.53 mM KCl, 0.53 mM MgCl and 11 mM D-glucose at pH 7.4 (adjusted with HCl).

For Brefeldin A (BFA) and H89 treatments, cells were incubated with 5 μg/ml BFA (Sigma-Aldrich, B5936) or 40 μM H89 (Merck, B1427), both dissolved in DMSO. Control cells were incubated in DMSO vehicle.

For overexpression of plasmids containing mNeongreen–Sec16A, mScarlet–Sec16A, GFP–Sec16B, Halo–Sec16A and YFP–Sec24D (see below for sources), INS-1 cells were transfected with Lipofectamine 2000 (Invitrogen, 11668019). DNA and Lipofectamine were mixed in OptiMEM (Gibco, 31985047) before being added to the cells. Cells were analyzed after 24–48 h of transfection.

For knockdown experiments, INS-1 cells were transfected in six-well plates with 5 nM siRNA using Lipofectamine RNAiMAX (Invitrogen, 13778030) and typically analyzed after 96 h. The siRNAs used were directed against Sec16A (rat) (Ambion, s137444) and Sec16B (rat) (Ambion, s137156).

### Animals

All experiments in Fig. S2 were approved by the DEC Dutch Animal Experiments Committee (Dier Experimenten Commissie), and performed in compliance with the institutional guidelines of University Utrecht, and conducted in agreement with Dutch law (Wet op de Dierproeven, 1996) and European regulations (Directive 2010/63/EU). The animal protocol has been evaluated and approved by the national CCD authority (license AVD1080020173404). Female pregnant Wistar rats were obtained from Janvier Labs (Le Genest-Satin-Isle, France), and embryos (both genders) at embryonic (E)18 stage of development were used for primary cultures of cortical neurons. The animals, pregnant females and embryos had not been involved in previous procedures.

### Primary culture of cortical neurons

To prepare primary cortical neurons, cortices from rat embryo brain at stage E18 were dissected and dissociated in trypsin for 15 min and then plated on coverslips coated with poly-L-lysine (37.5 μg/ml; Sigma-Aldrich, P2636) and laminin (1.25 μg/ml; Roche, 11243217001) at a density of 100,000 cells/well (12-well plate). The day of neuron plating corresponds to day *in vitro* 0 (DIV0). Neurobasal medium (NB) supplemented with 1% B27 (Gibco, 21103049), 0.5 mM glutamine (Gibco), 15.6 μM glutamate (Sigma) and 1% penicillin-streptomycin (Gibco) was used to maintain the neurons cultured under control temperature and CO_2_ conditions (37°C, 5% CO_2_).

To induce Sec body formation in cortical neurons, cells were incubated with either NB supplemented with 125 mM NaCl (NB125) or KRBm containing 0.7 mM Na_2_HPO_4_, 1.5 mM NaH_2_PO_4_, 30 mM NaHCO_3_, 167 mM NaCl, 4.53 mM KCl, 0.53 mM MgCl and 25 mM D-glucose at pH 7.4 (adjusted with HCl) for 3 h.

### DNA constructs

The following vectors were used: pCMV-mScarleti-C1, Addgene plasmid #85044 deposited by Dr Dorus Gadella (Bindels et al., 2017). pCMV-EGFP-C1 (obtained from Clontech). pCMV-EYFP-Sec24 (Stephens et al., 2000), Addgene plasmid #66614, and pCMV-EGFP-Sec16B (Budnik et al., 2011), Addgene plasmid #66607, deposited by Dr David Stephens. Strep-KDEL-Halo-Transferrin Receptor (TfR)-SBP (Halo-RUSH-TfR) (called RUSH-TfR in the text), Addgene plasmid #166905, deposited by Dr Jennifer Lippincott-Schwartz (Weigel et al., 2021); pF282-hEF1a-H2B-mNeonGreen-IRES-Puro×Tol2 was a gift from Dr Judith Klumperman (University Medical Center Utrecht, The Netherlands). Halo-Clathrin (Catsburg et al., 2022) was a gift from Dr Harold MacGillavry (Utrecht University, The Netherlands).

The following plasmids were generated in this study by Gibson assembly using NEBuilder^®^ HiFi DNA Assembly Master Mix (NEB, E2621 L). To obtain pCMV-mNeonGreen-Sec16A, we first generated the pCMV-EGFP-Sec16A construct. In detail, rat Sec16A (NM_001276417.1) was PCR amplified from a cDNA library obtained from INS-1-derived mRNA and inserted into pCMV-EGFP-C1 vector between the XhoI and KpI restriction sites. After generating pCMV-EGFP-Sec16A, the EGFP sequence from pCMV-EGFP-Sec16A was removed by restriction digest with AgeI and BsrGI and replaced with an mNeonGreen sequence. The mNeonGreen sequence was PCR amplified from the template hEF1a-H2B-mNeonGreen-IRES-Puro×Tol2, and a Kozak sequence was also added in front of mNeonGreen to increase the efficiency of translation. A 6-amino-acid flexible linker (SGLRSR) was introduced between mNeonGreen and the beginning of Sec16A by adding additional nucleotides to the cloning primers.

For pCMV-Halo-Sec16A, the construct was generated in a similar way as described above for pCMV-mNeonGreen-Sec16A. In detail, the EGFP sequence from pCMV-EGFP-Sec16A was removed by a digestion with AgeI and BsrGI and replaced with a Halo tag sequence. The Halo tag sequence was amplified by PCR from the template pHalo-Clathrin, and a Kozak sequence was added in front of the Halo tag sequence. A flexible linker of 7 amino acids (KSGLRSR) was flanked between the Halo tag sequence and the beginning of Sec16A by including extra nucleotides to the cloning primers.

For pCMV-mScarlet-Sec16A, rat Sec16A sequence was amplified by PCR from pCMV-EGFP-Sec16A and inserted into pCMV-mScarleti-C1 vector between the BamHI and BglII sites. A flexible linker of 6 amino acids (SGLSGS) was introduced between the mScarlet sequence and the beginning of Sec16A sequence by adding extra nucleotides to the cloning primers.

For pCMV-V5-Sec16A, a V5 sequence was amplified and inserted into pCMV-EGFP-Sec16A cut open with AgeI and BsrGI to replace the GFP sequence. A Kozak sequence and a flexible linker (GPKSGLRSR) were added in front and behind the V5 sequence, respectively.

For pCMV-V5-Sec16B, similarly, a V5 sequence was amplified and inserted into pCMV-BFP-Sec16B (generated in our laboratory) cut open with AgeI and HindIII. A Kozak sequence was added in front of V5, and a flexible linker (GPTNGSGSGS) was introduced behind V5 sequence. All primers used in this study are listed in Table S2.

### PCR

Sec16A-depleted cells, Sec16B-depleted cells or control cells grown in RPMI medium or incubated in KRBm for 4 h were spun down (4 min at 200 ***g***) and washed in PBS prior to RNA extraction. For each condition, RNA was extracted using the NucleoSpin Mini Kit (Macherey Nagel, 740955.50). The RNA concentrations were measured using a NanoDrop ND-1000 UV-Vis Spectrophotometer (Thermo Scientific). 1 μg RNA was used to synthesize cDNA using the GoScript Reverse Transcription System kit (Promega, A5000).

For each condition, a PCR was performed using Taq polymerase (Promega, M7841) and visualized on agarose gel to assess Sec16A and Sec16B. PCR primers used to detect Sec16A and Sec16B are listed in Table S2.

### Antibodies

For immunofluorescence, we used the primary antibodies rabbit anti-Sec16A (1:400; Bethyl, A300-648A), mouse anti-Sec13 (1:100; Santa Cruz Biotechnology, sc-514308), mouse anti-Sec24A (1:100; Santa Cruz Biotechnology, sc-517155), mouse anti-p115 (1:100; a gift from Martin Lowe, University of Manchester, UK), mouse anti-GM130 (1:500; a gift from Martin Lowe, University of Manchester, UK), rabbit anti-GRASP55 (1:1000; a gift from Micheal Bekier, University of Michigan, MI, USA) (Xiang and Wang, 2010), rabbit anti-GRASP65 (1:100; a gift from Fiona A. Barr, Mac Planck Insititut für Biochemie, Planegg, Germany) (Shorter et al., 1999), mouse-anti V5 (Invitrogen, R96025) and chicken anti-MAP2 (Abcam, ab5392). Donkey anti-rabbit-IgG Alexa Fluor 568 (1:500) (Invitrogen, A10042), goat anti-mouse-IgG Alexa Fluor 488 (1:500) (Invitrogen, A11001), donkey anti-mouse-IgG Alexa Fluor 555 (Invitrogen, A31570), donkey anti-rabbit-IgG Alexa Fluor 488 (Invitrogen, A21206), goat anti-mouse IgG2a Alexa Fluor 488 (Invitrogen, A21131), goat anti-mouse IgG1 Alexa Fluor 594 (Invitrogen, A21125), and goat anti-chicken-IgY Alexa Fluor 405 (Abcam, ab175675) were used as secondary antibodies. For STED imaging, we used rabbit anti-Sec16A and mouse anti-Sec13, followed by goat anti-rabbit-IgG CF594 antibody (1:1000) (Sigma-Aldrich, SAB4600110) and goat anti-mouse-IgG STAR635p (1:1000; Abberior, ST635P), respectively.

### Immunofluorescence

Cells were fixed in 4% paraformaldehyde in PBS (pH 7.4) for 15 min at room temperature. Then, cells were washed three times in PBS with 20 mM glycine (PBS-G) followed by permeabilization in PBS-G with 0.1% Triton X-100 for 10 min at room temperature. Subsequently, cells were washed three times with PBS-G and blocked in PBS-G with 0.5% fish skin gelatin (Sigma-Aldrich, G7041) for 20 min at room temperature. Next, cells were incubated with the primary antibody [in blocking buffer (PBS-G with 0.5% fish skin gelatin)] for 1 h at room temperature, followed by three times washing with blocking buffer, followed by three times washing with blocking buffer. Then, cells were incubated with the secondary antibody for 1 h at room temperature in the dark. Finally, cells were washed three times with PBS and coverslips were mounted on a microscope slide with Prolong Gold Antifade reagent with DAPI (Invitrogen, P36935).

### Immunoelectron microscopy

IEM was performed INS-1 cells in RPMI and after 4 h incubation in either KRBm or RPMI200. Cells were fixed in 2% PFA plus 0.2% glutaraldehyde, as previously described (Kondylis and Rabouille, 2003; van Donselaar et al., 2007). Ultrathin frozen sections were labeled with a monoclonal anti-Sec13 antibody as above, followed by a goat anti-mouse-IgG antibody and 15 nm Protein-A gold (PAG). Micrographs were collected on a JEM1010 (JEOL) equipped with a Veleta 2k×2k CCD camera (EMSIS, Münster, Germany).

### Microscopy and image acquisition

Fixed INS-1 cells were imaged on the laser scanning confocal microscope Leica SP8 or Zeiss LSM700. All images were acquired using a 63× oil immersion objective (NA 1.4).

For live-cell imaging experiments, we used an inverted microscope Nikon Eclipse Ti-E (Nikon), equipped with a Plan Apo VC 100× NA 1.40 oil objective (Nikon), a Yokogawa CSU-X1-A1 spinning disk confocal unit (Roper Scientific), a Photometrics Evolve 512 EMCCD camera (Roper Scientific) or Photometrics Prime BSI camera, and an incubation chamber (Tokai Hit) mounted on a motorized *XYZ* stage (Applied Scientific Instrumentation). To control all devices, MetaMorph (Molecular Devices) version 7.10.2.240 software was installed. For visualizing the Halo tag, cells were pre-incubated with Janelia Fluor^®^ 646 HaloTag^®^ ligand (100 nM) for 30 min, followed by a washing in RPMI-1640 medium prior to imaging. Coverslips were then mounted in a metal Ludin Chamber-Type I supplemented with the original medium from INS-1 cells, and were imaged in a Tokai Hit incubation chamber that maintains optimal temperature and CO_2_ (37°C and 5% CO_2_). To visualize different fluorescently tagged proteins, a laser channel was exposed for 200–300 ms, whereas for multi-color acquisition, different laser channels were exposed for 200–400 ms sequentially. Cells were then washed once and imaged in KRBm every 15 or 30 s for 30 min. Total time and intervals of imaging acquisition for each experiment are depicted in each legend for figure and/or legend for movies.

STED images were taken with the Leica SP83x microscope using a HC PL APO 100× NA 1.4 oil immersion STED WHITE objective. The 561 nm pulsed laser was used to excite CF594, and the 647 nm to excite STAR635p-labeled proteins. STAR635p was depleted with the 775 nm pulsed depletion laser, and CF594 was depleted with the 660 nm pulsed depletion laser. Images were taken for a single plane and were additionally subjected to deconvolution using Huygens deconvolution software. Deconvolution was performed using the classic maximum likelihood estimation (CMLE) deconvolution algorithm, with a maximum of 10 iterations and the signal-to-noise ratio (SNR) set at 7.

### Quantification of Sec16A remodeling and Sec body formation

Sec16A remodeling (and further Sec body formation) in INS-1 cells were quantified by using ImageJ (Schneider et al., 2012). In brief, a maximum z-projection was obtained from single z-plane images. Next, a threshold was set using the function ‘set threshold’ with various parameters depending on the experiment. Then the intensity and particle size were measured with the tool ‘Analyze particles’. The intensity and size of each particle from one condition were stacked in a large matrix and the total intensity per particle was calculated by multiplying the area by the intensity. We defined that a ‘large’ Sec body has a particle size of >0.3 μm and an intensity of >80% of the maximum intense particle. A ‘small’ Sec body was defined as having a particle size between 0.15 and 0.3 μm and an intensity of >75% of the maximum intense particle. The total intensity of all ‘small’ and ‘large’ Sec bodies were summed together to get the total intensity of all Sec bodies. The total intensity of Sec bodies per cell was represented by: (1) dividing the total intensity from all Sec bodies (respective to their criteria) by the total number of cells; or (2) mean values of total intensity of Sec bodies per cell from all cells.

For determining the degree of colocalization of two different proteins, the Mander's coefficient (ImageJ) was determined. Profile line plots were generated by tracing a line along a Sec body using the RGB Profile Plot plugin in ImageJ.

### RUSH assay to assess protein exit out of the ER

INS-1 cells (P60-P80) were conditioned in Dulbecco's modified Eagle medium (DMEM; Invitrogen), GlutaMAX Supplement, 10% FBS (Sigma-Aldrich, F7524) and 1% penicillin-streptomycin (Gibco, 15140122) for 3 days at 37°C and 5% of CO_2_ because RPMI contains high amounts of biotin preventing the retention of RUSH-TfR in the ER even in the absence of exogenous added biotin. Cells were plated to 50% confluency on coverslips in a 12-well plate 1 day before transfection. Per well, INS-1 cells were transfected with RUSH-TfR and Lipofectamine 2000 in OptiMEM. Subsequently, cells were washed and incubated in DMEM for 24 h. Cells were treated as indicated in the results. Unhooking of RUSH-TfR from the ER was mediated by the addition of D-biotin (Sigma-Aldrich, B4501-500MG) at 100 μM final concentration in DMEM and KRBm for 30 min prior to fixation. RUSH-TfR was visualized by staining cells with Janelia FluorX^®^ 554 HaloTag^®^ Ligand (100 nM).

### Statistical analysis

Data obtained at least from two independent experiments was processed and statistically analyzed using Excel and Graphpad Prism. A Mann–Whitney and Kruskal–Wallis test followed by a Dunn's multiple comparison test was performed for statistical analysis as indicated in figure legends. Significance was indicated as follows: ns, not significant; *P<0.05, **P<0.01, ***P<0.001. The assumption of data normality was evaluated using a D'Agostino-Pearson omnibus test.

## Supplementary Material

10.1242/joces.260294_sup1 Supplementary information
