# Supplementary material for: Stress-induced phase separation of ERES components into Sec bodies precedes ER exit inhibition in mammalian cells
Source: J Cell Sci. 2022 Dec 1;135(23):jcs260294. doi: 10.1242/jcs.260294 (PMC10112967; doi:10.1242/jcs.260294)
Supplement: Supplementary information [file joces-135-260294-s1.pdf]

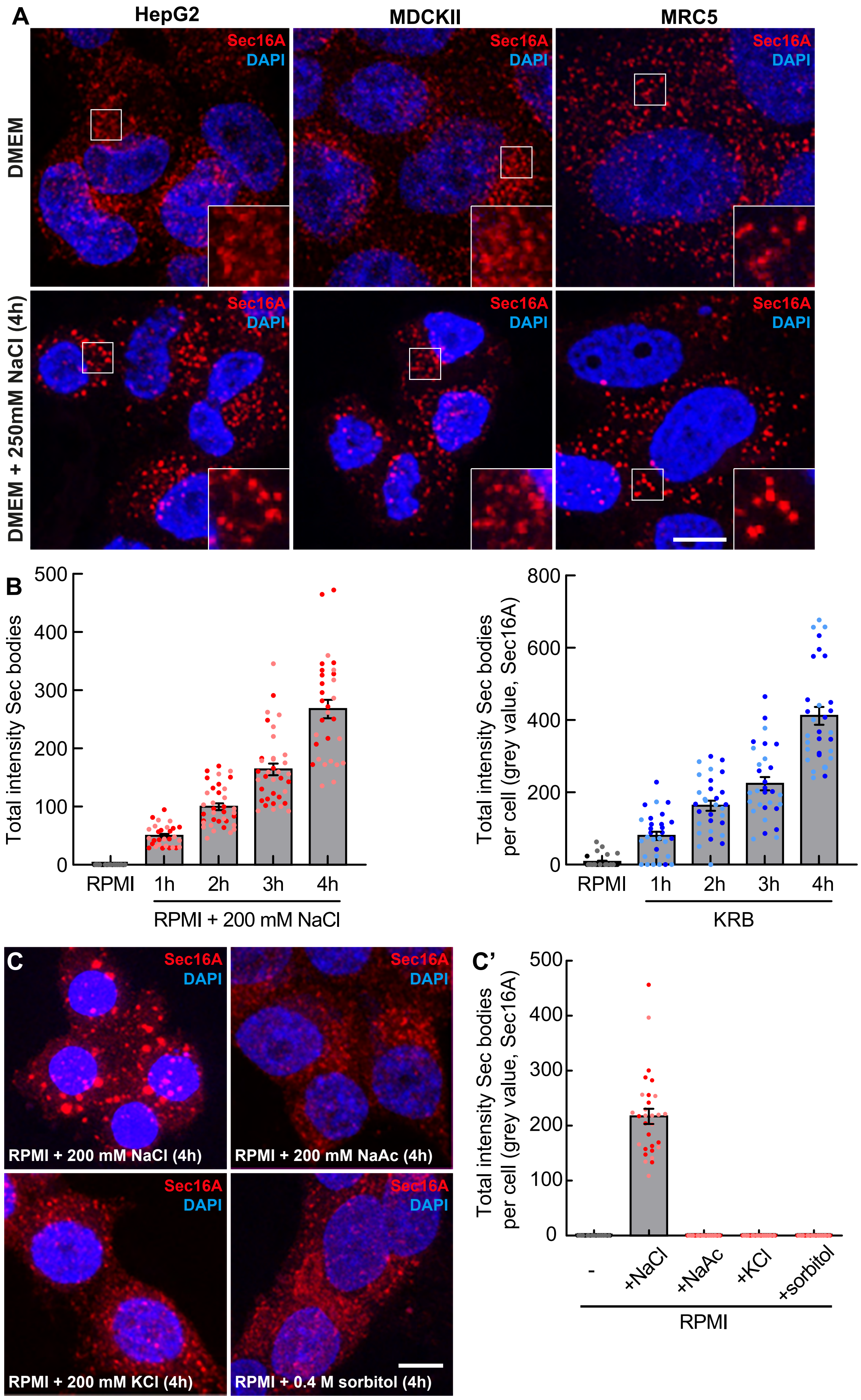

Journal of Cell Science • Supplementary information

Fig. S1. NaCl stress leads to the redistribution of Sec16A, mild in HepG2, MDCKII and MRC5 cells, and extensive in INS-1 cells leading to Sec body formation.
A: Immunofluorescence (IF) visualization of endogenous Sec16A in HepG2, MDCKII and MRC5 cells upon DMEM and DMEM+250 mM NaCl (4 h).
B: Bar plot depicting the total intensity of Sec bodies per cell in RPMI200 (left) and KRBm (right) over time (1-4 h) in INS-1 cells; N=2 experiments, n=29-36 cells.
C, C': IF visualization of Sec16A in INS-1 cells upon incubation in RMPI supplemented with either NaCl, or Na-Acetate or KCl (200 mM, 4 h), or 0.4 M sorbitol for 4 h (C). Note that large Sec16A-positive structures only form upon the addition of NaCl. Quantification of the total intensity of Sec16A-positive Sec bodies per cell (C'); N=2 experiments, n = 30 cells.
Scale bar: 10 µm (A) and (C).
Error bar: SEM (B) and (C').

Journal of Cell Science • Supplementary information

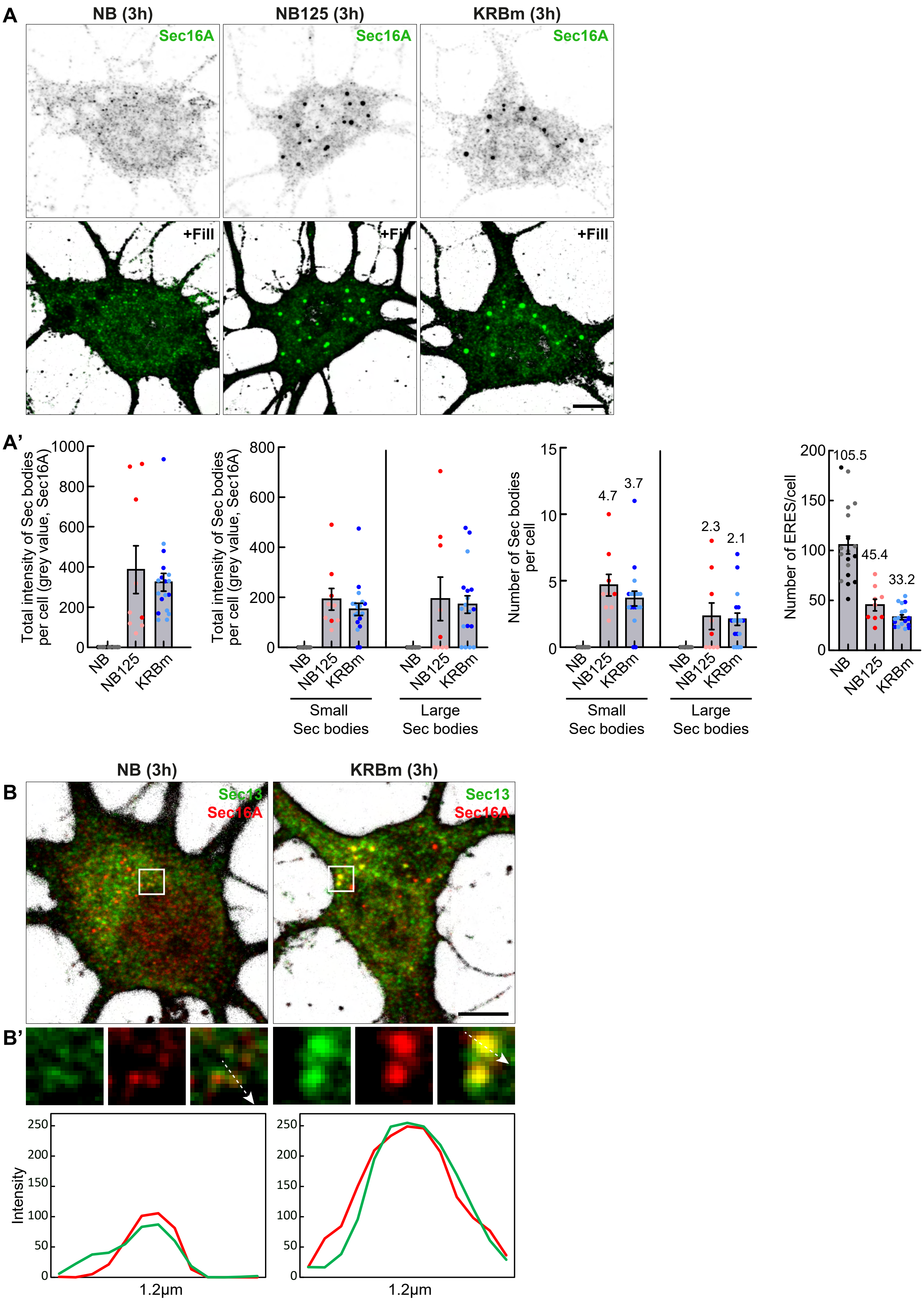
A
NB (3h)
NB125 (3h)
KRBm (3h)
Sec16A
+Fill
A'
Total intensity of Sec bodies per cell (grey value, Sec16A)
NB
NB125
KRBm
Small Sec bodies
Large Sec bodies
Number of Sec bodies per cell
4.7
3.7
2.3
2.1
Number of ERES/cell
105.5
45.4
33.2
B
Sec13
Sec16A
B'
Intensity
1.2μm

Fig. S2. Sec bodies form in neurons

A, A': Primary culture of rat cortical neurons treated with either Neurobasal (NB) neuronal media, KRBm or high salt (NB125, i.e., NB+125mM NaCl) for 3h and stained for endogenous Sec16A. Quantification of the total intensity of Sec16A-positive Sec bodies per cell (A'). N= 2 experiments, n= 9-18 cells

B, B': Neurons incubated with NB or KRBm for 3h and co-stained for Sec16A and Sec13. Intensity profile line in (B'). Notice co-distribution of two proteins within Sec bodies.

Scale bar: 5μm (A) and (B).

Error bar: SEM (A')

Journal of Cell Science • Supplementary information

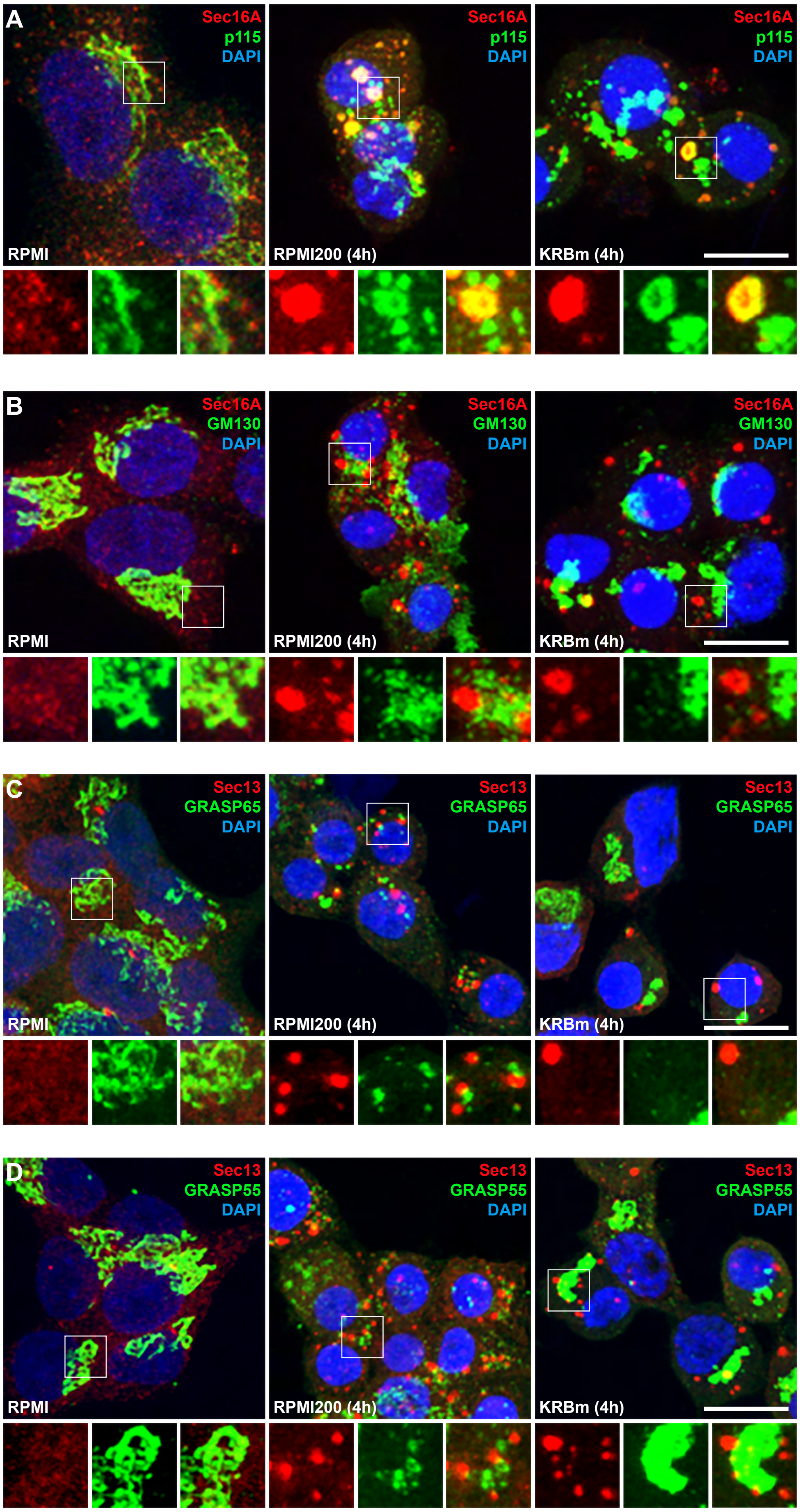

Journal of Cell Science • Supplementary information

Fig. S3. Sec16A-positive structures do not contain Golgi proteins

A: IF visualization of Sec16A and p115 in INS-1 cells upon incubation in RPMI, RPMI200 and KRBm (4 h). Note that a small fraction of p115 localizes in Sec16A positive structures.

B: IF visualization of Sec16A and the Golgi marker GM130 in INS-1 cells upon incubation in RPMI, RPMI200 and KRBm (4 h).

C: IF visualization of Sec13 and GRASP65 in INS-1 cells upon incubation in RPMI, RPMI200 and KRBm (4 h).

D: IF visualization of Sec13 and GRASP55 in INS-1 cells upon incubation in RPMI, RPMI200 and KRBm (4 h).

Scale bar: 10 µm (A-D)

Journal of Cell Science • Supplementary information

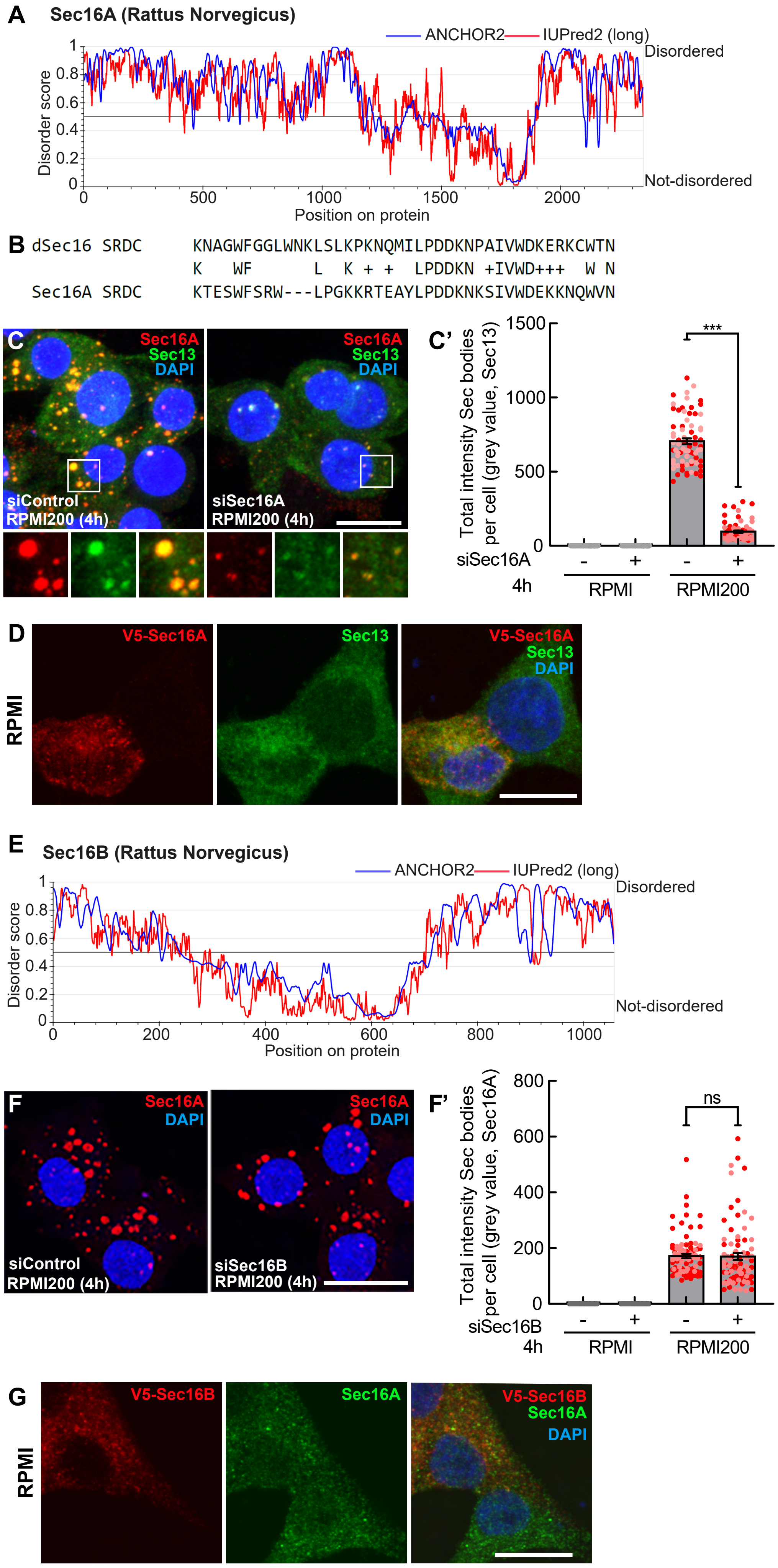

Journal of Cell Science • Supplementary information

Fig. S4. Additional information on Sec16A and Sec16B

A: Disordered regions in rat Sec16A as predicted by the database https://iupred2a.elte.hu/. A disorder score of 0.5 or higher is predicted as a disordered protein sequence.

B: SRDC in rat Sec16A. Top panel displays the reported SRDC region in *Drosophila* Sec16 (upper row) (Aguilera-Gomez et al., 2016) which is conserved in rat Sec16A (lower row). The overlap was found by protein sequence blasting using NCBI database. The SRCD is absent in rat Sec16B.

C, C': Immunofluorescence visualization of Sec16A and Sec13 in Sec16A depleted INS-1 cells incubated in RPMI200 for 4 h. Note that Sec body formation (marked by Sec13) is inhibited upon Sec16A depletion. Quantification of the total intensity of Sec bodies per cell (C'); N= 2 experiments, n = 65-73 cells

D: Representative images of V5-Sec16A in INS-1 transfected cells cultured in RPMI medium. Cells were labeled for V5-tag, DAPI, and for endogenous Sec13.

E: Disordered regions in rat Sec16B as predicted by the database https://iupred2a.elte.hu/.

F, F': Immunofluorescence visualization of Sec16A in Sec16B depleted INS-1 cells incubated in RPMI200 for 4 h (B). Note that Sec body formation (marked by Sec16A) is not inhibited upon Sec16B depletion. Quantification of the total intensity of Sec bodies per cell (C'); N= 2 experiments, n = 81-85 cells

G: Representative images of V5-Sec16B in INS-1 transfected cells cultured in RPMI medium. Cells were labeled for V5-tag, DAPI, and for endogenous Sec16A.

Scale bar: 10 μm (C), (D), (F) and (G).

Error bar: SEM (C') and (F'); ***p<0.001; ns – not significant.

Journal of Cell Science • Supplementary information

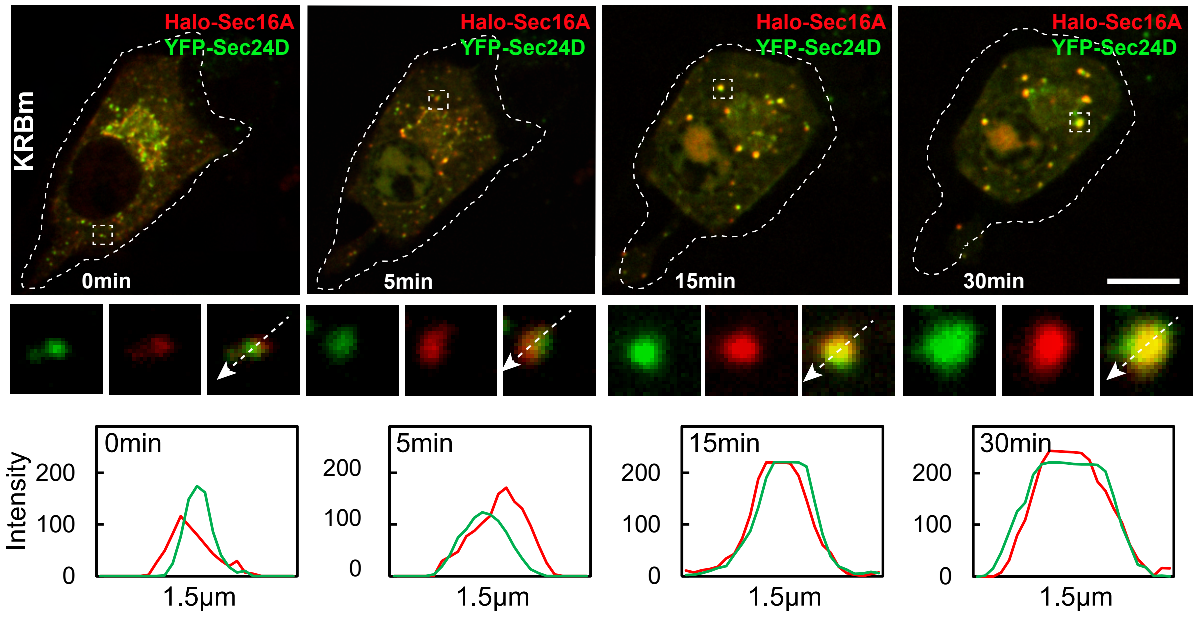

Fig. S5. ERES components are co-recruited into newly formed Sec bodies
Representative still images of time-points 0, 5, 15 and 30 min from a live cell expressing Halo-Sec16A and YFP-Sec24D and treated with KRBm during live-cell imaging. Cells were pre-incubated with the permeable Halo-646 dye prior to imaging. Single and merged channels of selected ERES and Sec body structures are shown, and intensity profile lines displaying co-distribution of Sec16A and Sec24D. See also *Movie S3*.
Scale bar: 5 µm

Journal of Cell Science • Supplementary information

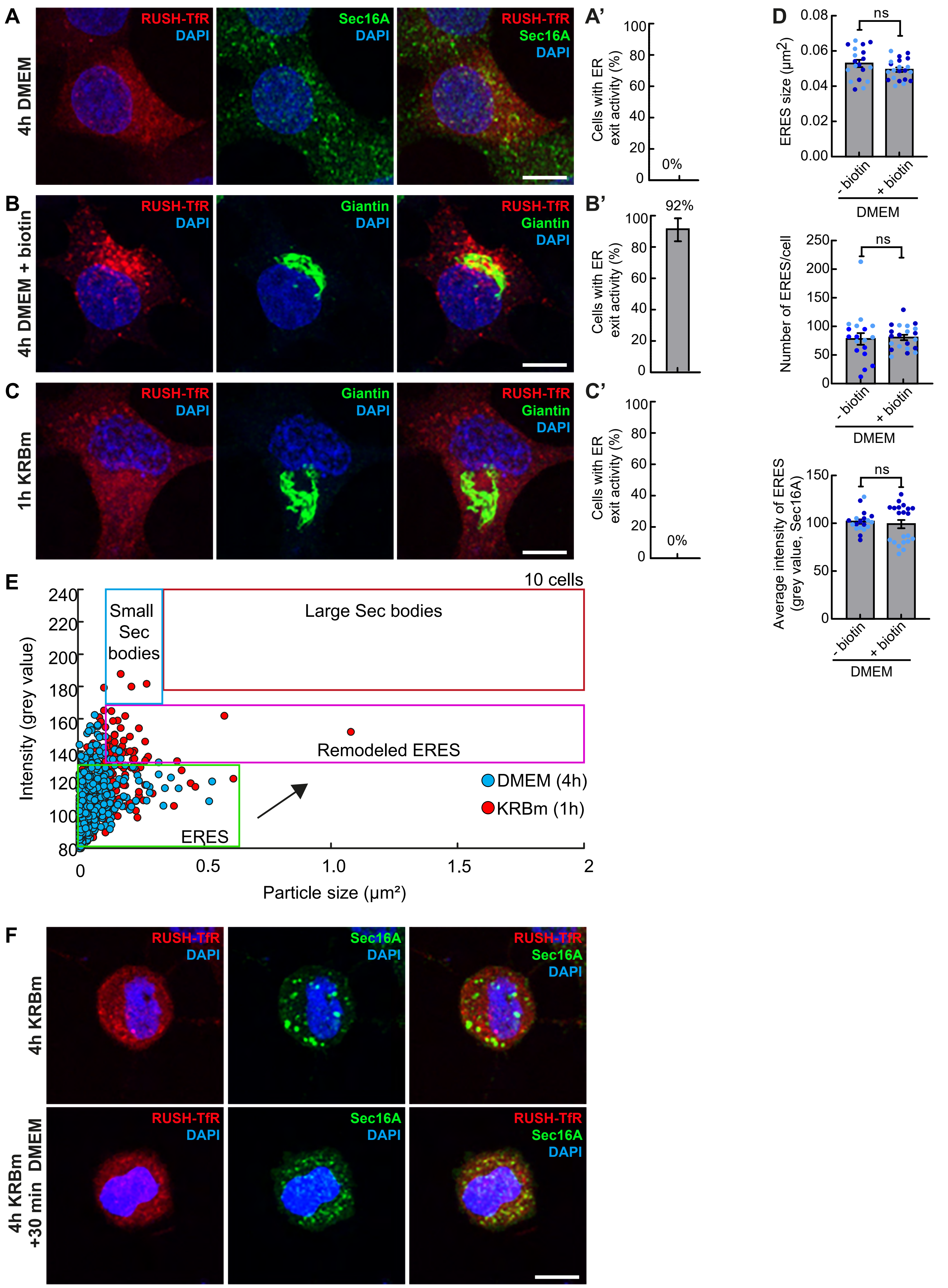

Journal of Cell Science • Supplementary information

Fig. S6. **Without addition of biotin RUSH-TfR is retained at the ER**
A, A': Visualization of RUSH-TfR and endogenous Sec16A in INS-1 cells in DMEM (4 h) without the addition of biotin. Quantification of the ER exit activity (A'); N= 2 experiments, n=13 cells
B, B': Visualization of RUSH-TfR and Giantin in cells cultured in DMEM (4 h) upon addition of biotin (30 min, 100 µM). Quantification of the ER exit activity (B'); N= 2 experiments, n=16 cells.
C, C': Visualization of RUSH-TfR and Giantin in cells in KRBm (1 h) without the addition of biotin. Quantification of the ER exit activity (C'); N= 2 experiments; n=15 cells.
D: Quantification of the average size (top), average number (middle) and average intensity of Sec16A- positive ERES per cell in cells maintained in DMEM with and without addition of biotin. N=2 experiments, n = 19-21 cells.
E: Scatterplot depicting Sec16A foci size and intensity upon incubation in DMEM (blue dots) and 1 h KRBm (red dots) of 10 INS-1 cells. Note that foci become larger and more intense after 1 h of KRBm incubation (magenta box, remodeled ERES).
F: Visualization of RUSH-TfR and endogenous Sec16A in cells incubated in KRBm for 4 h followed by 30 min or 1 h in DMEM without biotin. Note that the Sec bodies have dissolved but that RUSH-TfR is retained in the ER.
Scale bar: 10 µm
Error bar: SEM (A'-C') and (D); ns – not significant.

Journal of Cell Science • Supplementary information

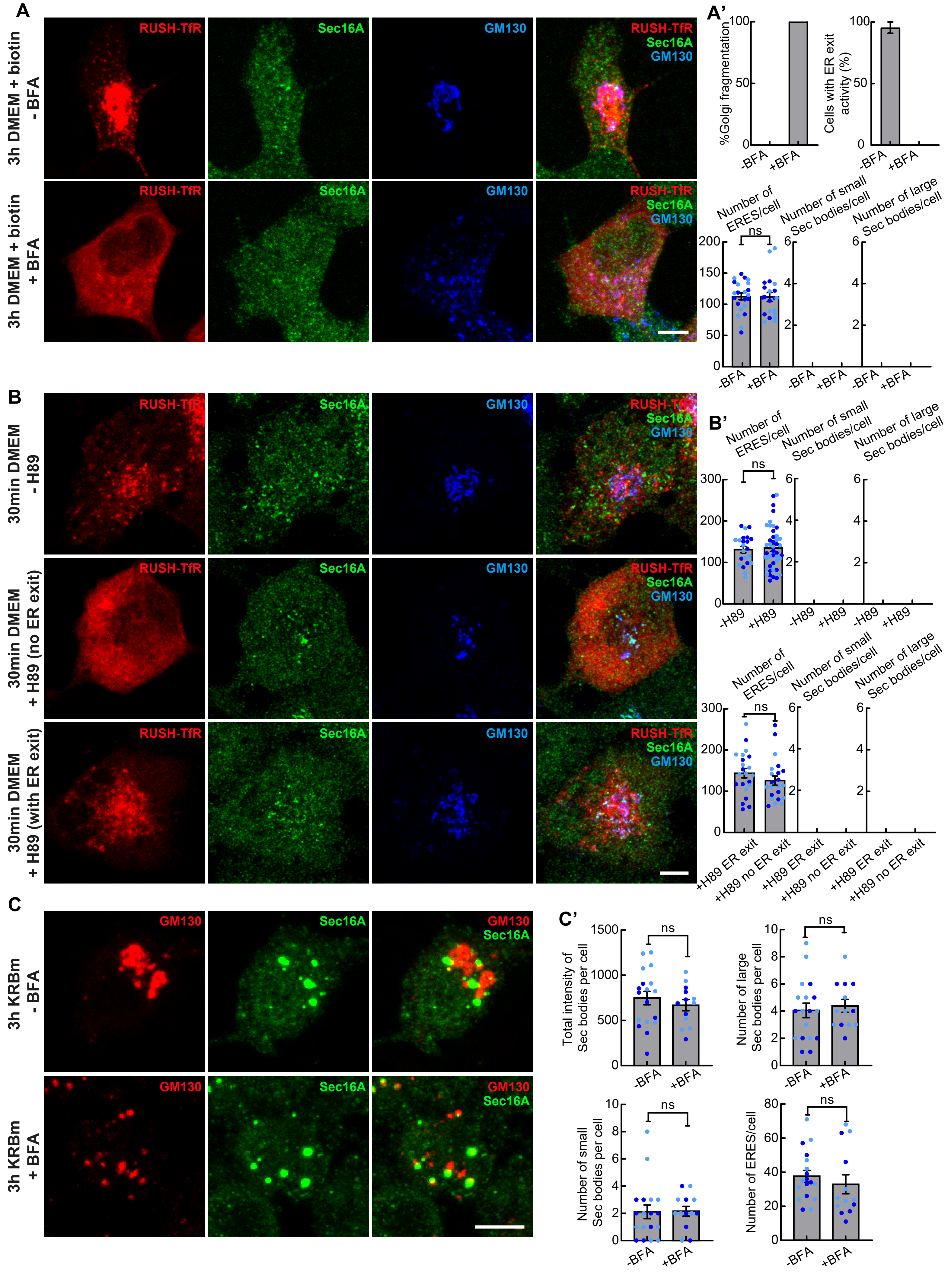

Journal of Cell Science • Supplementary information

Fig. S7. ER exit inhibition is neither a driving nor a contributing factor in Sec body formation

A, A': Visualization of RUSH-TfR (red), Sec16A (green) and the Golgi marker GM130 (blue) in INS-1 cells in DMEM incubated or not with BFA for 3 h in DMEM plus biotin during the last 30 min of incubation. Quantification of the percentage of cells displaying Golgi fragmentation, ER exit activity, as well as their number of ERES, and small and large Sec bodies in (A'). Note that BFA treatment does not promote Sec body formation. N= 2 experiments; n=21-22 cells.

B, B': Visualization of RUSH-TfR (red), Sec16A (green) and the Golgi marker GM130 (blue) in INS-1cells in DMEM pre-incubated or not with H89 for 5min followed by co-incubation of H89 plus biotin during the last 25 min. Quantification of number of ERES, and small and large Sec bodies in (B') comparing control cells and H89 treated cells (top) and H89-treated cells with ER exit (47%) and no ER exit (53%) (bottom). Note that H89 does not promote Sec body formation in cells displaying ER exit inhibition. N= 2 experiments; n = 22-44 cells.

C, C': IF visualization of Sec16A (green) and GM130 (red) in INS-1 cells treated with BFA for 30 min before and during the 3 h of KRBm incubation. Quantification of the total intensity of Sec bodies, number of small and large Sec bodies, and number of ERES. Note that they are not altered by BFA treatment upon stress (C'). N= 2 experiments; n=13-18 cells.

Scale bar: 5μm

Error bar: SEM (A'), (B'), and (C'); ns – not significant.

Journal of Cell Science • Supplementary information

Table S1. (Related to Fig. 7)

	ERES	Small Sec bodies	Large Sec bodies
**Average number per structure**			
DMEM	97.08 ± 28.59	0	0
KRB 1h	53.61 ± 26.9	0.5 ± 0.87	0.28 ± 0.61
KRB 3h (ER exit)	53.75 ± 23.46	2.58 ± 2.13	2.13 ± 2.13
KRB 3h (no ER exit)	27.38 ± 13.72	2.34 ± 1.62	3.94 ± 2.02
KRB 4h	22.84 ± 13.67	2.88 ± 3.13	5.00 ± 1.96
**Average size per structure**			
DMEM	0.04 ± 0.01	N/A	N/A
KRB 1h	0.08 ± 0.04	0.21 ± 0.03	0.50 ± 0.18
KRB 3h (ER exit)	0.06 ± 0.02	0.20 ± 0.02	0.72 ± 0.33
KRB 3h (no ER exit)	0.08 ± 0.04	0.21 ± 0.03	0.71 ± 0.27
KRB 4h	0.085 ± 0.05	0.22 ± 0.02	0.82 ± 0.29
**Average intensity per structure**			
DMEM	89.74 ± 9.83	N/A	N/A
KRB 1h	103.02 ± 6.99	147.36 ± 27.09	152.87 ± 31.75
KRB 3h (ER exit)	137.18 ± 18.50	182.87 ± 17.2	196.83 ± 11.66
KRB 3h (no ER exit)	151.3 ± 31.91	192.12 ± 25.42	203.21 ± 23.16
KRB 4h	143.79 ± 21.79	193.68 ± 15.15	204.87 ± 15.48
**Total intensity of Sec bodies per cell (small plus large Sec bodies)**			
DMEM			0
KRB 1h			40.65 ± 75.4
KRB 3h (ER exit)			407.81 ± 369.6
KRB 3h (no ER exit)			730.16 ± 477.4
KRB 4h			930.10 ± 496.4

Table S2.

Primer	Sequence
pCMV-EGFP-Sec16A_F	5'-GGACTCAGATCTCGAATGCAGCCACCACCTCAG-3'
pCMV-EGFP-Sec16A_R	5'-GATCCCGGGCCCGCGCTAGTTCAGTGCCGCATATTTTCTTTGG-3'
pCMV-mNeonGreen-Sec16A_F	5'-CGTCAGATCCGCTAGCGCTAGCCACCATGGTGAGCAAGGGCGAG-3'
pCMVmNeonGreen-Sec16A_R	5'-CGAGATCTGAGTCCGGACTTGTACAGCTCGTCCATGCC-3'
pCMVHalo-Sec16A_F	5'-CGTCAGATCCGCTAGCGCTAGCCACCATGGCAGAAATCGGTACTGG-3'
pCMVHalo-Sec16A_R	5'-CGAGATCTGAGTCCGGACTTGCCGGAAATCTCGAGCGTG-3'
pCMV-mScarleti-Sec16A_F	5'-GCTGTACAAGTCCGGACTCAGCGGCAGCATGCAGCCACCACCTCAG-3'
pCMV-mScarleti-Sec16A_R	5'-TCAGTTATCTAGATCCGGTGCTAGTTCAGTGCCGCATATTTTC-3'
pCMV-V5-Sec16A_F	5'-CGTCAGATCCGCTAGCGCTAACTGCCACCATGGGTAAG-3'
pCMV-V5-Sec16A_R	5'-CGAGATCTGAGTCCGGACTTGGGCCCCGTAGAATCGAG-3'
pCMV-V5-Sec16B_F	5'-CGTCAGATCCGCTAGCGCTAACTGCCACCATGGGTAAG-3'
pCMV-V5-Sec16B_R	5'-TACCGCTGCCGCTACCATTAGTGGGCCCCGTAGAATCGAG-3'
PCR alpha-tubulin_F	5'-TTTCTACCAGGCAGACGATGAGC-3'
PCR alpha-tubulin_R	5'-CCAGCAATGGAGTGACACAGT-3'
PCR Sec16A_F	5'-TGGAAGCCAAGGTGGTAAGC-3'
PCR Sec16A_R	5'-CAGGGTTGTAAAAGGGCACA-3'
PCR Sec16B_F	5'-AGGAGAAAGAGTGGCAAGGC-3'
PCR Sec16B_R	5'-TAGGCCTCCAAGTTGCTGAC-3'

Journal of Cell Science • Supplementary information

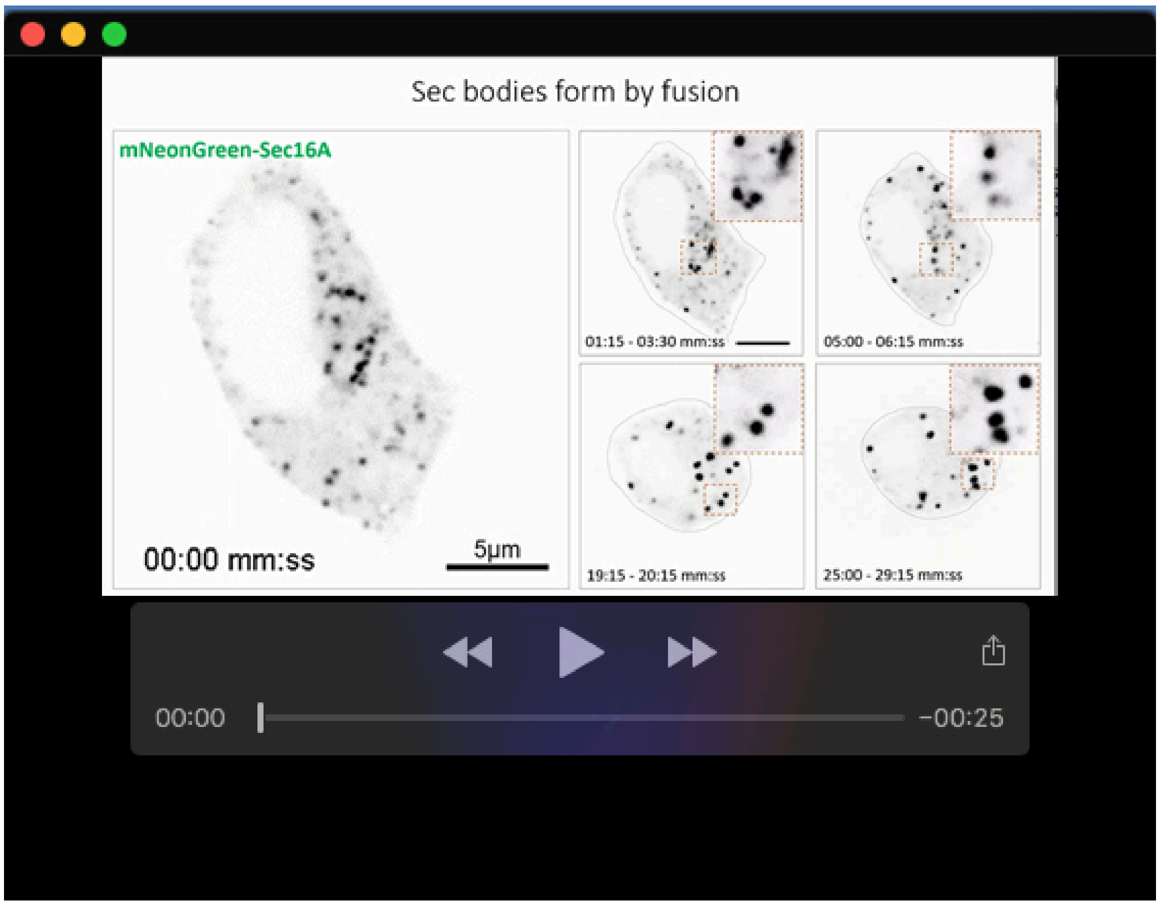

Movie 1. (related to Fig. 6). Sec bodies form by fusion. INS-1 cells expressing mNG-Sec16A were incubated with KRBm and immediately imaged every 15 seconds for 30 minutes. Right panels are zoom-in images of Sec bodies undergoing fusions at indicated time points.

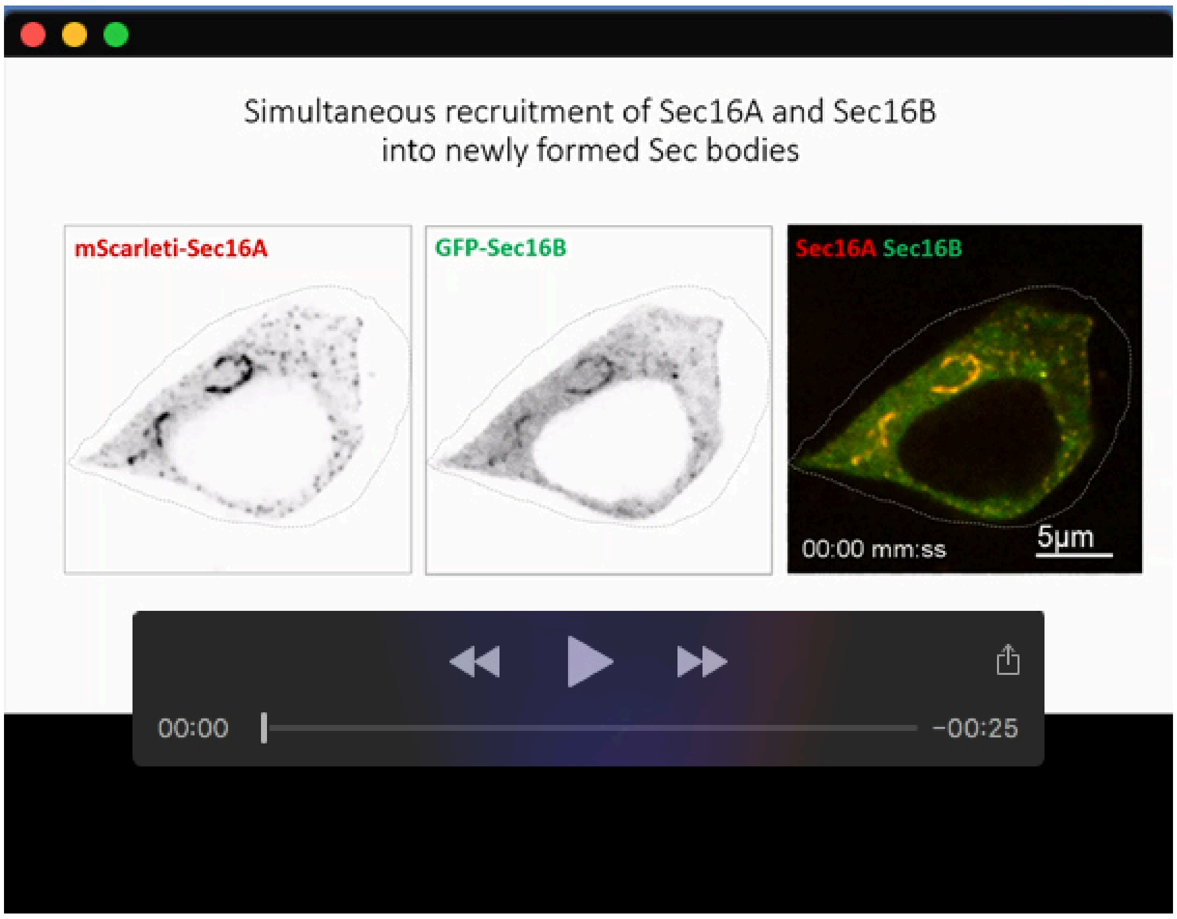

Movie 2. (related to Fig. 6). Simultaneously recruitment of Sec16A and Sec16B into newly formed Sec bodies. INS-1 cells co-expressing mScarlet-Sec16A and GFP-Sec16B were incubated with KRBm and immediately imaged every 30 seconds for 30 minutes. Notice co-distribution of proteins in reorganized structures undergoing fusion events.

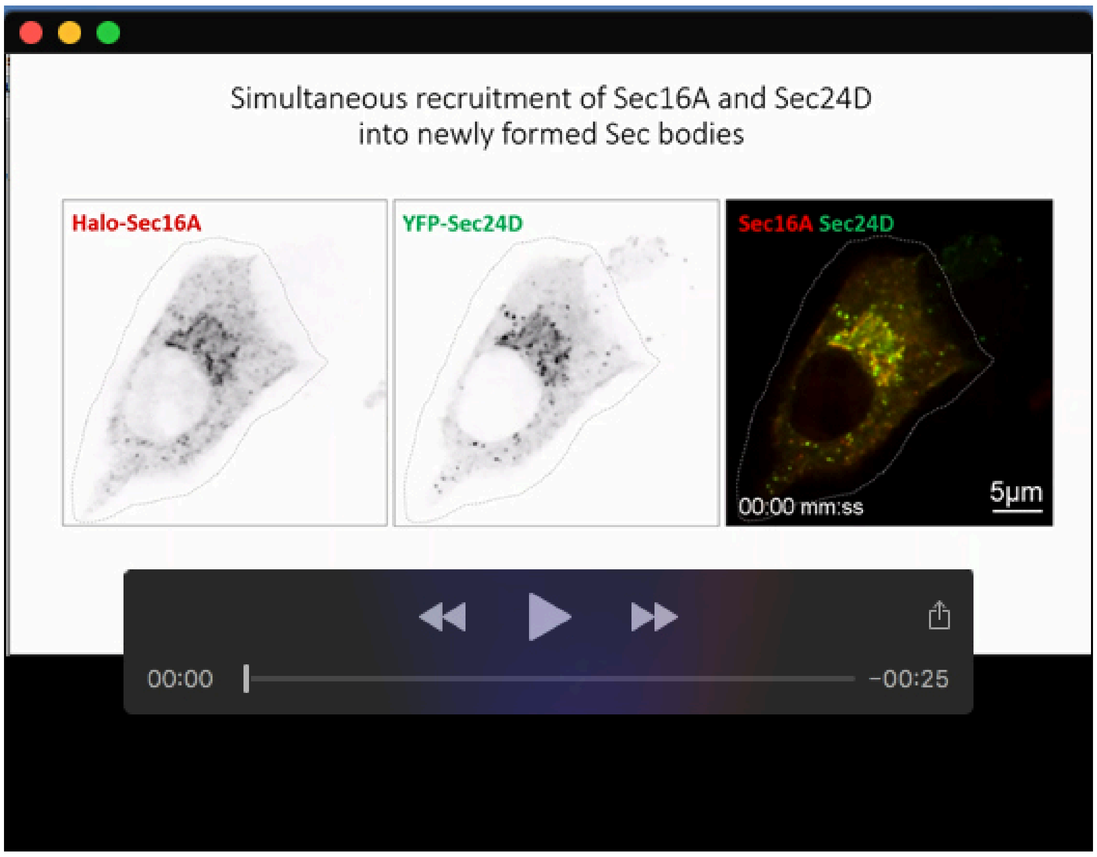

Movie 3. (related to Fig. 6). Simultaneously recruitment of Sec16A and Sec24D into newly formed Sec bodies. Sec bodies in INS-1 cells co-transfected with Halo-Sec16A and YFP-Sec24D. Cells were pre-incubated with a permeable Halo-646 dye prior KRB treatment during imaging every 30 seconds for 30 minutes. Notice co-distribution of proteins in reorganized structures undergoing fusion events.

Journal of Cell Science • Supplementary information
