# Supplementary material for: Stress-induced phase separation of ERES components into Sec bodies precedes ER exit inhibition in mammalian cells
Source: J Cell Sci. 2022 Dec 1;135(23):jcs260294. doi: 10.1242/jcs.260294 (PMC10112967; doi:10.1242/jcs.260294)
Supplement: Supplementary file 1 [file joces-135-260294_review_history.pdf]

Stress-induced phase separation of ERES components into Sec bodies precedes ER exit inhibition in mammalian cells

Wessel van Leeuwen, Dan T. M. Nguyen, Rianne Grond, Tineke Veenendaal, Catherine Rabouille and Ginny G. Farías
DOI: 10.1242/jcs.260294

Editor: David Stephens

Review timeline

Submission to Review Commons:	4 March 2022
Original submission:	30 May 2022
Editorial decision:	31 May 2022
First revision received:	1 September 2022
Editorial decision:	3 October 2022
Second revision received:	25 October 2022
Accepted:	27 October 2022

Reviewer 1

Evidence, reproducibility and clarity

Van Leeuwen et al. analyze and characterize Sec bodies in mammalian cells. The lab has discovered previously Sec bodies in Drosophila, which are membraneless organelles containing ER exit site (ERES) and COPII components and which form under specific stress conditions. They find that Sec bodies can also be formed at least in a rat cell line that highly secretes insulin, INS-1 under osmotic stress or a combination of amino acid starvation and osmotic stress. They establish that the ERES component Sec16A but not its isoform Sec16B is a driver of Sec body formation while still being a constituent of this organelle. Finally, the authors aimed to elucidate the relationship between Sec bodies and ERES and whether block ERES drives Sec body formation or whether Sec body formation causes a block in ER exit.

While most of the data provided is convincing, there are some shortcomings in particular with the RUSH experiments. The data on the regulation of ERES numbers by Sec bodies is not fully convincing. The authors could either tone down their conclusions or would need to provide more and stronger evidence.

Specific points:

Major points:
- The authors talk about remodeling of ERES into large Sec16A positive structures. It is unclear what the authors mean with remodeling. Sec16A is re-routed, changes its localization, but nothing more. There are no other ERES components are shown. Clearly, this is premature and an overinterpretation at this point to conclude that ERES goes into Sec16A positive structures, if the only marker that is shown in Sec16A. So, either the authors also show other ERES components (i.e combine Fig 1+2) or they need to re-write this part to stick with what they can conclude: Sec16A changes its localization under these specialized conditions.
- The number of independent experiments performed for various experiments is not clear. In some cases, where it is stated, it's only two experiments. This not up to the standard; at least three independent experiments should be provided.

© 2022. Published by The Company of Biologists under the terms of the Creative Commons Attribution License (https://creativecommons.org/licenses/by/4.0/).

- The authors use the Mander's coefficient for co-localization analysis. They should give both directions as the COPII components might still be at the ER and allow some ER exit, even if the corralling protein Sec16A is reduced.
- Sec16A is not quantitatively in Sec bodies. This is particularly obvious in Fig. 5C and D. Is the remainder of Sec16A still ER-associated and supports COPII vesicle budding?
- It is unclear why the incubation times vary from experiment to experiment (except for Fig. 6 and 7). They authors may want to provide a better rational why sometimes 1 hr, 2 hrs or 4 hrs are used
- The issue with Figure 7 is manifold: The standard definition used by the authors for Sec bodies by size and fluorescence intensity is not useful in this instance. In using the RUSH system, the ERES will increase in size and hence also the Sec16 staining (compare Fig. 7A and Fig S6A). In Panel A. there are no Sec bodies counted, but they are similar in size to what they count as small Sec bodies. It seems as if they just arbitrarily decided what is an ERES and what is a Sec body. This is important because in panel 7B, most of the TfR has left the ER and there are many large Sec16A structures that might be small Sec bodies, but they are not counted as such. These data are very ambiguous. The author would need to use a ERES marker that does not go into Sec bodies, such as Sar1. This applies than also for the rest of the figure and makes it impossible to solidly conclude that Sec bodies regulate the number of ERES. Also trying to correlate number of ERES/cell with Total intensity of Sec bodies/cell is not something easily digestible. A fair comparison would be the 'relocalization'/changes in fluorescence intensities from ERES to Sec bodies, but for this a clear distinction of Sec bodies and ERES would be needed. A possibility would be that the authors take out Figure 7 altogether and refrain from making this conclusion

Minor points:

- The summary of the results at the end of the introduction is almost as long as the entire introduction and too many details are given. The introduction should be streamlined.
- P. 4, 3rd paragraph: The authors did not test a large set of Golgi proteins, but rather a set of large Golgi proteins.
- P.7, 2nd last paragraph. Why should TfR ever get in a membraneless compartment? TfR contains a transmembrane domain and is normally an obligate dimer (i.e. 2 TMDs/complex). This statement does not make sense and should be removed.

Significance

The authors provide convincing evidence for Sec bodies also at least in one mammalian cell line. Therefore, their data imply that the larger dataset previously published by the group using Drosophila S2 cells might be generalisable for some mammalian cell lines with respect to osmotic stress response (+ aa starvation). They nicely show different roles for the ERES scaffold proteins Sec16A and Sec16B in the process of Sec body formation.
The paper is a nice addition to the still relative scarce literature on secretion/membrane traffic under stress.

reviewer expertise: membrane traffic, yeast and mammalian cells

Reviewer 2

Evidence, reproducibility and clarity

This study examines the formation of a stress-responsive membraneless compartment in mammalian cells that regulates secretion. Building on previous work in Drosophila S2 cells, which identified SEC bodies that sequester COPII trafficking machinery in response to nutrient and salt stress, the current work surveys mammalian cells for similar effects, finding that INS-1 cells also activate this pathway. Through a series of elegant, well-performed and thorough imaging analyses, the authors convincingly show that the mammalian system closely mirrors that of Drosophila. The findings are clearly presented, convincing and appropriately interpreted.
I have several relatively minor comments that might improve the manuscript for the reader.
1. Figure 2: do small SEC bodies have less co-localization with COPII proteins than larger ones? This might suggest progression from an early, small Sec16-positive structure to larger COPII-positive structures. I was also curious as to whether there was a difference in co- localization intensity

© 2022. Published by The Company of Biologists under the terms of the Creative Commons Attribution License (https://creativecommons.org/licenses/by/4.0/).

(Mander coefficient) between inner and outer coat components in early versus late (i.e. small versus large) SEC bodies.
2. Figure 3A: in the left panel, the right hand cluster of Sec13 labelling seems to be associated with membrane structures. Is this remnant ERES?
3. Figure 7 D/E: I found the initial description of these experiments confusing. The authors should state clearly that D and E represent the two different populations of cells under the same incubation conditions. It was also unclear to me how the conclusion was reached from this experiment that SEC body formation preceded ER exit inhibition. To my mind, there is simply a correlation between SEC body size/extent and secretion defects. This should be spelled out more clearly. Similarly, the method for defining what corresponds to an ERES versus a small SEC body should be more clearly stated in the results here so that the reader can understand the distinction and subsequent quantification.

Significance

This study represents a significant advance in that it reveals a conserved stress-response pathway that acts in professional secretory cells. There is little new mechanistic insight (eg. the singling pathways or PTMs that contribute to SEC body formation) but that does not detract from the significance of the findings. The work will be of interest to researchers in the protein trafficking field, as well as those interested more broadly in liquid-liquid phase separation and membraneless organelles.

Reviewer 3

Evidence, reproducibility and clarity

This group previously identified Sec bodies as membraneless organelles that form in Drosophila S2 cells upon NaCl stress or amino acid starvation to encapsulate components of ER exit sites (ERES). Here they demonstrate that Sec bodies also form under similar stress conditions in highly secretory mammalian INS-1 cells. A key component of mammalian Sec bodies is Sec16A, while the smaller Sec16B protein is a component of Sec bodies but is not a driver of their formation. The results indicate that sequestration of Sec16A inhibits ER exit by reducing the number of ERES below a critical threshold. This inhibition is readily reversible when the stress is relieved. The conclusion is that Sec16A-driven formation and dissolution of Sec bodies is a mechanism to regulate ER exit in response to environmental conditions.

I have only one minor issue with the data. Knockdown of Sec16A inhibits Sec body formation, but presumably ER exit will also be blocked. If so, how can indirect effects of the Sec16A knockdown be ruled out? This result is suggestive of a crucial role for Sec16A in Sec body formation but is not definitive, and the interpretation should be appropriately cautious.

Significance

The main takeaway message is that the Sec body formation initially discovered in invertebrates also occurs in at least some mammalian cells. As such, the novelty of the study is only moderate. On the other hand, the finding that Sec body formation is a widespread phenomenon is a significant advance. Moreover, this work extends the prior data from Drosophila with new insights obtained by characterizing the composition, dynamics, and functions of mammalian Sec bodies. The experiments are exceptionally clean and elegant, and they are presented well. For these reasons, the paper will be of broad interest to researchers interested in membrane traffic and membraneless organelles.

© 2022. Published by The Company of Biologists under the terms of the Creative Commons Attribution License (https://creativecommons.org/licenses/by/4.0/).

Author response to reviewers' comments

We thank the reviewers for their overall very positive comments and questions, and their support of the manuscript.
Below, we have addressed all their points indicating the textual changes, clarifications and experimental approaches we will take to revise the manuscript. The experiments we propose to do are highlighted in grey and are summarised in a section "experimental plan". In green, we have highlighted the Figures for Rev where we have addressed some of the points raised by the reviewers.

Reviewer #1 (Evidence, reproducibility and clarity)
Van Leeuwen et al. analyze and characterize Sec bodies in mammalian cells. The lab has discovered previously Sec bodies in Drosophila, which are membraneless organelles containing ER exit site (ERES) and COPII components and which form under specific stress conditions. They find that Sec bodies can also be formed at least in a rat cell line that highly secretes insulin, INS-1 under osmotic stress or a combination of amino acid starvation and osmotic stress. They establish that the ERES component Sec16A but not its isoform Sec16B is a driver of Sec body formation while still being a constituent of this organelle. Finally, the authors aimed to elucidate the relationship between Sec bodies and ERES and whether block ERES drives Sec body formation or whether Sec body formation causes a block in ER exit.

While most of the data provided is convincing, there **are some shortcomings in particular with the RUSH experiments. The data on the regulation of ERES numbers by Sec bodies is not fully convincing.** The authors could either tone down their conclusions or would need to provide more and stronger evidence.

Specific points
Major points
Rev1.1: The authors talk about remodeling of ERES into large Sec16A positive structures. It is unclear what the authors mean with remodeling. Sec16A is re-routed, changes its localization, but nothing more. There are no other ERES components are shown. Clearly, this is premature and an overinterpretation at this point to conclude that ERES goes into Sec16A positive structures, if the only marker that is shown in Sec16A. So, either the authors also show other ERES components (i.e combine Fig 1+2) or they need to re-write this part to stick with what they can conclude: Sec16A changes its localization under these specialized conditions.
>> We understand the concern of the reviewer that mentioning the stress-induced changes in Sec16A distribution as "ERES remodeling" in Figure 1 is premature without showing other ERES components. This comes in Figure 2. We will therefore mention this figure 2 earlier.

The reviewer suggests combining Figures 1 and 2. This could make the resulting figure crowded, especially with the incorporation of new data that we have obtained the nanoscale distribution of ERES proteins using STED. This shows that Sec bodies (small and large) contain both Sec16 and Sec13 (see Figure 1 for Rev). This new data will be extended and added to the edited manuscript.

Rev1.2: The number of independent experiments performed for various experiments is not clear. In some cases, where it is stated, it's only two experiments. This not up to the standard; at least three independent experiments should be provided.
>> We will add information of number of independent experiments in all Figures.
In general, we have performed two independent experiments counting 30-50 cells for many of these experiments. For most of the data showed in the manuscript, the results are consistent between cells and between independent experiments, and with small variation for conditions tested. Furthermore and very importantly, the effects we observed are almost 'black and white". for instance reversion, and hexanediol (Figure 3). These effects of these treatments are over 90%, and we have not deemed necessary to spend time and resources to perform a third experiment to confirm this significant effect.

Figure 1 displays the quantification of representative experiments. Importantly this quantitative analysis has been collected from many experiments performed independently, and these results have been recapitulated in many subsequent experiments shown as controls used to compare other conditions.

© 2022. Published by The Company of Biologists under the terms of the Creative Commons Attribution License (https://creativecommons.org/licenses/by/4.0/).

About the Sec16A and B depletion (Figures 4A-B'; Fig 5A-B'), the question is not whether we can efficiently and reproducibly deplete these proteins. We can. The question is whether their depletion has effect on Sec body formation, which we have monitored by analyzing many cells on 2 independent experiments for each Sec16A and Sec16B. There were hardly any variations between experiments and again, the effects were very large. When Sec16A is depleted to over 85%, Sec body formation is inhibited by 90%. When Sec16B is depleted to over 80%, Sec body formation is not inhibited. Furthermore, Sec16A depletion was monitored, cell by cell using immunostaining: The more depleted, the less Sec bodies formed.

For live cell imaging (Figure 6B), we have quantified 11 transfected cells from 2 independent experiments. Data acquisition and analysis is extensive and very time consuming. We show individual cells in graph, with similar temporal kinetics for Sec body fusion events. In additional experiments, similar results were obtained, fast Sec body formation / fusion events with same Sec16A marker together with Sec16B (Figure 6C), or with Sec24D (Figure 6D).

For RUSH (Figure 7), we have performed 3 experiments, two with Sec16 as a Sec body marker (quantification shown), and one with Sec13 (data not shown), leading to the same conclusion.

Overall, our experimental conditions have large effects or no-effects and without large variation between cells and independent experiments. Statistics differences are clear when comparing groups from at least two independent experiments. Furthermore, different markers and techniques have been used to show remodeling of ERES into Sec bodies. In this regard, we respectfully trust that repeating experiments for all the figures that that have been generated from only two independent experiments, is unnecessary. This would be a large amount of time and resources to confirm what we have significantly shown in the manuscript.

Instead, we will focus on repeating experiments to generate Mander's coefficient M1 and M2 (Figure 2) (**Rev1 and 2**), provide further quantification of the number of ERES/Sec bodies (small and large) in the RUSH system (Figure 7) (**Rev1 and 2**) and strengthen the correlation between Sec body formation and blockage in ER exit (**Rev 1,2 and 3**).

Rev1.3: The authors use the Mander's coefficient for co-localization analysis. They should give both directions as the COPII components might still be at the ER and allow some ER exit, even if the corralling protein Sec16A is reduced.
>> We will provide M1 and M2 Mander's values, for the entire cell as it is presented in Figure 2, but also structure per structures by using ROI Mander's. See also **Rev2.1**.

Rev1.4: Sec16A is not quantitatively in Sec bodies. This is particularly obvious in Fig. 5C and D. Is the remainder of Sec16A still ER-associated and supports COPII vesicle budding?
Rev1.5: It is unclear why the incubation times vary from experiment to experiment (except for Fig. 6 and 7). They authors may want to provide a better rational why sometimes 1 hr, 2 hrs or 4 hrs are used
>> We agree with the reviewer that endogenous Sec16A is not quantitatively recruited in Sec bodies in Figure 5D. This is because, in this experiment, the KRBm incubation has only been performed for 30min, a too short incubation to promote the robust formation of Sec bodies in wild type cells. In fact, it takes between 2 and 4h to induce a quantitative Sec body formation under this condition. The reason we have chosen such a short KRBm incubation is to assess whether overexpression of Sec16A or B accelerate the process of Sec body formation. A long incubation would diminish the dynamic range of the measurement (efficacy and kinetics). With using 30min, we can state that the Sec16A overexpression promotes Sec body formation (when compared to non-transfected cells), whereas this is not the case for Sec16B. These results are in line with the depletion approach showing that Sec16A, but not Sec16B, is a driver in Sec body formation. We will provide a time-course of Sec body formation for KRB (as we did for high salt) to better explain the rational with regards to selecting this short incubation time.

Rev1.6a: The issue with Figure 7 is manifold: The standard definition used by the authors for Sec bodies by size and fluorescence intensity is not useful in this instance. In using the RUSH system, the ERES will ssincrease in size and hence also the Sec16 staining (compare Fig. 7A and Fig S6A).
>>As requested by the reviewer, we have now compared Figure 7A and Supp Figure 6A. To us, they are undistinguishable, only with a minor variation in size and intensity of Sec16A-positve structures when releasing cargo from the ER with biotin (when compared to control where no biotin is added). We therefore respectfully disagree that cargo release significantly enlarges the ERES, at least in

© 2022. Published by The Company of Biologists under the terms of the Creative Commons Attribution License (https://creativecommons.org/licenses/by/4.0/).

this experiment. To investigate this further, we will perform a systematic quantitative analysis of these two conditions.

Rev1.6b: In Panel A. there are no Sec bodies counted, but **they are similar in size** to what they count as small Sec bodies. It seems as if they just arbitrarily decided what is an ERES and what is a Sec body. This is important because in panel 7B, most of the TfR has left the ER and there are many large Sec16A structures that might be small Sec bodies, but they are not counted as such. These data are very ambiguous.
The reviewer hints to the fact that we have not provided a clear distinction between an ERES and a small Sec body. For clarification, ERES are usually fainter in endogenous Sec16 staining (or other COPII subunit) by immunofluorescence, and irregular (not round) (see Figure 2A for Rev). When we analyze their number in cells grown in RPMI, we find that there are about 100 ERES per cell, a figure similar to what is published in the field for other cells. When using the quantification, we designed (Figure 1A' of the manuscript), the ERES cluster to the bottom left corner of the graph (now marked by a green BOX in (Figure 2B for Rev) in a size ranging from 0.01 to 0.14 um and an intensity below 70% of the maximal intensity particle. By IEM, they are characterized, as they have been before in many cells, by the presence of an ER cup "limiting" a collection of membrane, buds, tubules and vesicles marked by Sec13 (Figure 2C for Rev).

Sec bodies are structures different from ERES, and they have not been arbitrary assigned. They are the structures visible in stressed INS1 cells with KRBm and RPMI200 for 4h. The IF intensity of Sec16 (and all markers used) is higher than ERES and they are larger and tend to adopt a round shape (See Figure 2A, B for Rev). Small Sec bodies have a size of 0.15-0.3um2 and an intensity > 75% of the max intense particle. Large Sec bodies have a size larger than 0.3um2 with an intensity above 80% of the max intense particle. By IEM, they are very different from ERES, as they are more electron dense (Figure 2C for Rev). They do not contain membrane and vesicles, yet their Sec13 immunogold density appears equal or higher than at ERES. This is in total agreement with the increased intensity observed by IF.

Of note, not all Sec16 and Sec13 (or other COPII subunit) coalesce in Sec bodies upon stress. We still observe the presence of ERES upon stress (about 25 per cells) as shown in Figure 3A (top corner) in the original manuscript, and in Figure 2A for Rev (white circle).
Given these definitions and the careful observation and quantifications of images over many experiments, we can safely distinguish ERES from small Sec bodies by immunofluorescence. We have used these definitions in all experiments, including Figure 7A. We will provide this data in the revised manuscript to highlight the clear distinction between ERES and Sec bodies.

We agree that expressing Sec body formation as 'total intensity Sec body per cell' that includes both small and large Sec body populations (intensity*size) is confusing. For a clearer interpretation of the data in Figure 7, we will modify the graph to display the number and intensities of both small and large Sec body populations, separately for each time point. This will nail the point that Sec body formation precedes inhibition of ER exit. Of note, and even we have lacked clarity of this aspect, the important parameter in this inhibition is the number of ERES left that can function in ER export (see point **Rev1.6c** and **Rev2.3c**).

Rev1.6c: The author would need to use a ERES marker that does not go into Sec bodies, such as Sar1. This applies than also for the rest of the figure and **makes it impossible to solidly conclude that Sec bodies regulate the number of ERES.**
>> In order to determine whether ERES are still present, we appreciate the suggestion of analyzing an ERES component that does not go into Sec bodies, First, given the definition given above, ERES are still present after 4h stress (about ¼ of the RPMI condition). We will analyze this with other ERES markers. Second, as suggested, we have tested Sar1-GFP in INS1 cells. In control conditions, Sar1-GFP is not strictly at ERES sites, but also along ER membrane, similar to what has been shown in previous studies (Yorimitsu & Sato, Mol Biol Cell, 2012; Kurokawa, et al., J Cell Sci., 2016). It therefore makes it difficult to assess the number of remaining ERES. We will try to locate a good antibody to detect endogenous Sar1.

Furthermore, we have found that Sar1-GFP is also re-localized from ER into undefined structures that are close to, but not overlapping with, Sec bodies (see Figure 3 for Rev) [NOTE: We have removed a Figure which was provided for the Reviewers in confidence]. This is similar to what has been observed in *Drosophila* cells (Zachargianni et al, 2014). This Sar1 behavior in stress

© 2022. Published by The Company of Biologists under the terms of the Creative Commons Attribution License (https://creativecommons.org/licenses/by/4.0/).

appears to be a different biological process altogether that would require much more work, outside the scope of this present study.

Taken together, given that Sec16A/B, Sec23, Sec24 and Sec13 are present in INS-1 Sec bodies (as shown in different Figures of our manuscript), recruited in a concomitant manner as Sec16A, we propose that without any of these components essential for COPII coat formation that are recruited to Sec bodies, less ERES would be functional in ER export resulting in its inhibition.

Rev1.6d: Also trying to correlate number of ERES/cell with Total intensity of Sec bodies/cell is not something easily digestible. A fair comparison would be the 'relocalization'/changes in fluorescence intensities from ERES to Sec bodies, but for this a clear distinction of Sec bodies and ERES would be needed. A possibility would be **that the authors take out Figure 7 altogether and refrain from making this conclusion**.

>>We agree that Figure 7, where we have not separated small and large Sec bodies, is confusing. For a clearer interpretation of the data, we will change 'total intensity Sec body per cell' graph to display number and intensities of both small and large Sec body populations, separately. The data are already generated.

We take the opportunity here to stress that what critically controls secretion is NOT the amount/number of Sec bodies formed (large and small), but how much ERES are left to support ER exit.

We propose that a certain threshold of ERES number (above a quarter) is needed to support proper secretion measured by RUSH.

Of note, it is correct that these remaining ERES could be altered in other ways, thus not supporting ER exit for reasons others than their depletion of Sec16 and COPII subunits. We will mention this possibility in the revision.

Minor points:

- The summary of the results at the end of the introduction is almost as long as the entire introduction and too many details are given. The introduction should be streamlined.

>> We will shorten the summary at the end of the introduction as requested.

- P. 4, 3rd paragraph: The authors did not test a large set of Golgi proteins, but rather a set of large Golgi proteins.

>> We will rephrase this.

- P.7, 2nd last paragraph. Why should TfR ever get in a membraneless compartment? TfR contains a transmembrane domain and is normally an obligate dimer (i.e. 2 TMDs/complex). This statement does not make sense and should be removed.

>> Indeed, Sec bodies are not sealed by a membrane (Figure 3). As they derive from ERES components and form where ERES were present, they could potentially have encapsulated several COPII coated membrane derived from the ER and therefore containing cargo.

However, upon re-examination of Sec body profiles by EM, we do not find consistent evidence for the presence of such membrane contained within Sec bodies (although as already mentioned in the original manuscript, they are often in the close proximity to ER membrane). Although this corroborates the finding that we do not find any co-localization of TfR with Sec bodies, we will remove the statement as it is unnecessarily confusing.

Significance:

The authors provide convincing evidence for Sec bodies also at least in one mammalian cell line. Therefore, their data imply that the larger dataset previously published by the group using Drosophila S2 cells might be generalisable **for some mammalian cell lines** with respect to osmotic stress response (+ aa starvation). They nicely show different roles for the ERES scaffold proteins Sec16A and Sec16B in the process of Sec body formation. The paper is a nice addition to the still relative scarce literature on secretion/membrane traffic under stress.

>>Thank you. We also want to point that we have established Sec body formation in other mammalian cells, i.e., neurons, and we will add these data to this manuscript. Sec body formation upon stress is therefore generalizable to more than 1 mammalian cell type.

© 2022. Published by The Company of Biologists under the terms of the Creative Commons Attribution License (https://creativecommons.org/licenses/by/4.0/).

Reviewer #2 (Evidence, reproducibility and clarity):

This study examines the formation of a stress-responsive membraneless compartment in mammalian cells that regulates secretion. Building on previous work in Drosophila S2 cells, which identified SEC bodies that sequester COPII trafficking machinery in response to nutrient and salt stress, the current work surveys mammalian cells for similar effects, finding that INS-1 cells also activate this pathway. Through a series of elegant, well-performed and thorough imaging analyses, the authors convincingly show that the mammalian system closely mirrors that of Drosophila. The findings are clearly presented, convincing and appropriately interpreted.
I have several relatively minor comments that might improve the manuscript for the reader.

Rev2.1. Figure 2: do small SEC bodies have less co-localization with COPII proteins than larger ones? This might suggest progression from an early, small Sec16-positive structure to larger COPII-positive structures. I was also curious as to whether there was a difference in co-localization intensity (Mander coefficient) between inner and outer coat components in early versus late (i.e. small versus large) SEC bodies.
>> We appreciate the request for clarification from the reviewer. In Figure 2, we show the overlap of two endogenous proteins per cell. To distinguish the marker's overlap in small and large Sec bodies, we will quantify this overlap per structures (small and large Sec bodies) by using ROI Mander's.

In addition, we have got better understanding of the distribution of these proteins at the nanoscale level by STED super-resolution microscopy for endogenous Sec16A and Sec13, showing that in both small and large Sec bodies, Sec16 forms a "shell" around Sec13 (see Figure 1 for Rev). We will perform further similar experiments to solidify these preliminary experiments.

Last, we would like to point to the reviewer's attention that we have performed (and incorporated) dual live cell imaging using tagged Sec16A/Sec16B, Sec16A/Sec24D (Figure 5E, 6C-D and suppl Figure S5) showing in kinetic detail the concomitant co-distribution of ERES proteins during the remodeling from ERES to small and large structures. The coalescence of the ERES markers occur at the same time as Sec16A.

Rev2.2. Figure 3A: in the left panel, the right-hand cluster of Sec13 labelling seems to be associated with membrane structures. Is this remnant ERES?
>>The reviewer is correct. The mentioned structure it is an ERES that have not been remodeled. It has the same feature as the ERES in cells grown in RPMI (see Figure 2C for Rev). This will be mentioned in a clearer manner.

Rev2.3a: Figure 7 D/E: I found the initial description of these experiments confusing. The authors should state clearly that D and E represent the two different populations of cells under the same incubation conditions.
>>We apologize as we thought the text was clear in this regard. The two categories of cells (treated for 3h KRBm) are made by sorting the cells that display ER exit from those that do not (using RUSH).
In the cells where ER exit can be visualized (TfR out of the ER), the number of ERES is around 50. In the cells where no ER exit can be visualized (TfR in the ER), the number of ERES is low (24). This number is similar to cells incubated for 4h in KRBm where no ER exit is visualized (95% of the cells). We will explain this better in the manuscript.

Rev2.3b: It was also unclear to me how the conclusion was reached from this experiment that SEC body formation preceded ER exit inhibition. To my mind, **there is simply a correlation between SEC body size/extent and secretion defects.** This should be spelled out more clearly.
>>The reviewer is correct. What we propose is that there is a correlation between ER exit inhibition and the number of functional ERES are left that are still competent for transport. We propose that Sec body formation titrates away Sec16 and COPII subunits away from ERES, making them unfunctional.

What we say is that Sec body formation precedes the inhibition of ER exit and is therefore not a consequence of ER inhibition. To show this more firmly, we will perform an experiment with BFA that blocks traffic in the early secretory pathway and ultimately blocks ER exit. In Drosophila cells, we have clearly shown that BFA incubation does not lead to Sec body formation (Zacharogianni et al, 2014).

© 2022. Published by The Company of Biologists under the terms of the Creative Commons Attribution License (https://creativecommons.org/licenses/by/4.0/).

We have pushed this claim in mentioning “causality”, i.e., that Sec body formation causes this inhibition by titrating away key components. We will mellow this statement and stick to “precede” as we agree that there might be other causes to this inhibition, modifications, other effects of the stress to other part of the machinery etc.

Rev2.3c: Similarly, the method for defining what corresponds to an ERES versus a small SEC body should be more clearly stated in the results here so that the reader can understand the distinction and subsequent quantification.
The reviewer hints to the fact that we have not provided a clear distinction between an ERES and a small Sec body. For clarification, ERES are usually fainter in endogenous Sec16 staining (or other COPII subunit) by immunofluorescence, and irregular (not round) (see Figure 2A for Rev). When we analyze their number in cells grown in RPMI, we find that there are about 100 ERES per cell, a figure similar to what is published in the field for other cells. When using the quantification we designed (Figure 1A’ of the manuscript), the ERES cluster to the bottom left corner of the graph (now marked by a green BOX in Figure 2B for Rev) in a size ranging from 0.01 to 0.14 um and an intensity below 70% of the maximal intensity particle. By IEM, they are characterized, as they have been before in many cells, by the presence of an ER cup “limiting” a collection of membrane, buds, tubules and vesicles marked by Sec13 (Figure 2C for Rev).

Sec bodies are different structures and they have not been arbitrary assigned. They are the structures visible in stressed INS1 cells with KRBm and RPMI200 for 4h. The IF intensity of Sec16 (and all markers used) is higher than ERES and they are larger and tend to adopt a round shape (See Figure 2A, B for Rev). Small Sec bodies have a size of 0.15-0.3um2 and an intensity > 75% of the max intense particle. Large Sec bodies have a size larger than 0.3um2 with an intensity above 80% of the max intense particle. By IEM, they are very different from ERES, as they are more electron dense (Figure 2C for Rev). They do not contain membrane and vesicles, yet their Sec13 immunogold density appears equal or higher than at ERES This is in total agreement with the increase intensity observed by IF.

Of note, not all Sec16 and Sec13 (or other COPII subunit) coalesce in Sec bodies upon stress. We still observe the presence of ERES upon stress (about 25 per cells) as shown in Figure 3A (top corner) in the original manuscript, and in Figure 2A for Rev (white circle).

Given these definitions and the careful observation and quantifications of images over many experiments, we can safely distinguish ERES from small Sec bodies by immunofluorescence. We have used these definitions in all experiments, including Figure 7A. We will provide this data in the revised manuscript to highlight the clear distinction between ERES and Sec bodies.

We agree that expressing Sec body formation as ‘total intensity Sec body per cell’ that includes both small and large Sec body populations (intensity*size) is confusing. For a clearer interpretation of the data in Figure 7, we will modify the graph to display the number and intensities of both small and large Sec body populations, separately for each time point. This will nail the point that Sec body formation precedes inhibition of ER exit. Of note, and even we have lacked clarity of this aspect, the important parameter in this inhibition is the number of ERES left that can function in ER export (see point **Rev1.6b**).

Significance:
This study represents a significant advance in that it reveals a conserved stress-response pathway that acts in professional secretory cells. There is little new mechanistic insight (e.g. the singling pathways or PTMs that contribute to SEC body formation) but that does not detract from the significance of the findings. The work will be of interest to researchers in the protein trafficking field, as well as those interested more broadly in liquid-liquid phase separation and membraneless organelles.
>>Thank you. Of note, if it is correct that Sec body formation occurs quantitatively in INS1 cells, a clear ERES remodeling upon the stress described in the manuscript is observed in all the other mammalian cell type we tested. The resulting structures could be named “small Sec body”, the large ones being indeed rarer. We have perhaps provided a negative impression in the beginning of our manuscript in dismissing these other cell lines. We have focused on the INS-1 cell line where the response is the strongest, thus allowing us to provide a quantitative aspect to our study. We

© 2022. Published by The Company of Biologists under the terms of the Creative Commons Attribution License (https://creativecommons.org/licenses/by/4.0/).

will rephrase this in the revised manuscript. Furthermore, we have mentioned that Sec bodies form in neurons and we will show these data in the revision.

Reviewer #3 (Evidence, reproducibility and clarity):
This group previously identified Sec bodies as membraneless organelles that form in Drosophila S2 cells upon NaCl stress or amino acid starvation to encapsulate components of ER exit sites (ERES). Here they demonstrate that Sec bodies also form under similar stress conditions in highly secretory mammalian INS-1 cells.
>>Of note, if it is correct that Sec body formation occurs quantitatively in INS1 cells, a clear ERES remodeling upon the stress described in the manuscript is observed in all the other mammalian cell type we tested. The resulting structures could be named “small Sec bodies”, the large ones being indeed rarer. We have perhaps provided a negative impression in the beginning of our manuscript in dismissing these other cell lines. We have focused on the INS-1 cell line where the response is the strongest, thus allowing us to provide a quantitative aspect to our study. We will rephrase this in the revised manuscript. Furthermore, we have mentioned that Sec bodies form in neurons and we will show these data in the revision.

A key component of mammalian Sec bodies is Sec16A, while the smaller Sec16B protein is a component of Sec bodies but is not a driver of their formation. The results indicate that sequestration of Sec16A inhibits ER exit by reducing the number of ERES below a critical threshold. This inhibition is readily reversible when the stress is relieved. The conclusion is that Sec16A-driven formation and dissolution of Sec bodies is a mechanism to regulate ER exit in response to environmental conditions.

Rev3.1: I have only one minor issue with the data. Knockdown of Sec16A inhibits Sec body formation, but presumably ER exit will also be blocked. If so, how can indirect effects of the Sec16A knockdown be ruled out? This result is suggestive of a crucial role for Sec16A in Sec body formation but is not definitive, and the interpretation should be appropriately cautious.
>> We appreciate the question from the reviewer. Indeed, Sec16a depletion severely reduce secretion (Bhattacharyya & Glick, MBoC, 2007) and in this regard, it is possible that this ER exit inhibition is the cause of Sec body formation. However, there are two arguments against this: First, Sec16B depletion also severely reduces secretion and ER exit (Bhattacharyya & Glick, MBoC, 2007), yet Sec body formation is not affected. Second, at least in Drosophila, cell incubation with BFA prior to KRB stress did not change Sec body formation. WE conclude that a block in ER exit (and trafficking in the early secretory pathway) does not impede on Sec body formation. This will be explained better in the manuscript and the experiment with BFA will be performed in INS1 cells.

Significance:
The main takeaway message is that the Sec body formation initially discovered in invertebrates also occurs in at least some mammalian cells. As such, the novelty of the study is only moderate. On the other hand, the finding that Sec body formation is a widespread phenomenon is a significant advance. Moreover, this work extends the prior data from Drosophila with **new insights obtained by characterizing the composition, dynamics, and functions of mammalian Sec bodies**. The experiments are **exceptionally clean and elegant**, and they are presented well. For these reasons, the paper will be of **broad interest to researchers interested in membrane traffic and membraneless organelles**.
>>Thank you.

Experimental plan for revision of #RC-2022-01343.
The reasoning for each of these experiments is outlined in the rebuttal and they derive from the concern of the reviewers.

- Provide statistical data of significance for each experiment.

- Perform the quantitation of Sec body formation upon KRBm incubation over time 1-4h (**Rev 1.5**).

- Extend and analyse STED microscopy endogenous Sec16/Sec13. (**Rev1.1; Rev2.1**). See Figure for Rev 1

© 2022. Published by The Company of Biologists under the terms of the Creative Commons Attribution License (https://creativecommons.org/licenses/by/4.0/).

- Calculate M1 and M2 Mander's coefficient for small and large Sec bodies (**Rev1.3; Rev2.1**).
- Perform the quantitative comparison between +biotin -biotin in cells in DMEM (Figure 7A and Suppl Fig S6A) (**Rev1.6a**).

- In Figure 1, indicate the clear distinction between an ERES and a small Sec body (**Rev1.6b; Rev2.3c**) as a supplementary figure similar to Figure 2 for Rev, including IF and IEM **(Rev1.6b; Rev2.2).** See Figure for Rev 2.

- Display number and intensities of small and large Sec bodies in Figure 7 (**Rev1.6b; Rev2.3c**). Repeat experiment and quantification for n=3.

- Test a commercial antibody against Sar1 (**Rev1.6c**).

- Perform the BFA experiment prior to KRBm (**Rev2.3; Rev3.1**). will also be associated to RUSH.

- Show Sec bodies in neurons **(Rev1-2-3).**

- Textual additions, clarification.

3. Description of the revisions that have already been incorporated in the transferred manuscript

We have not yet incorporated changes to the manuscript but prepare figures for Reviewers as part of the point-by-point rebuttal. Below the Figures for reviewers.

4. Description of analyses that authors prefer not to carry out

Figure 1 for editor and reviewer

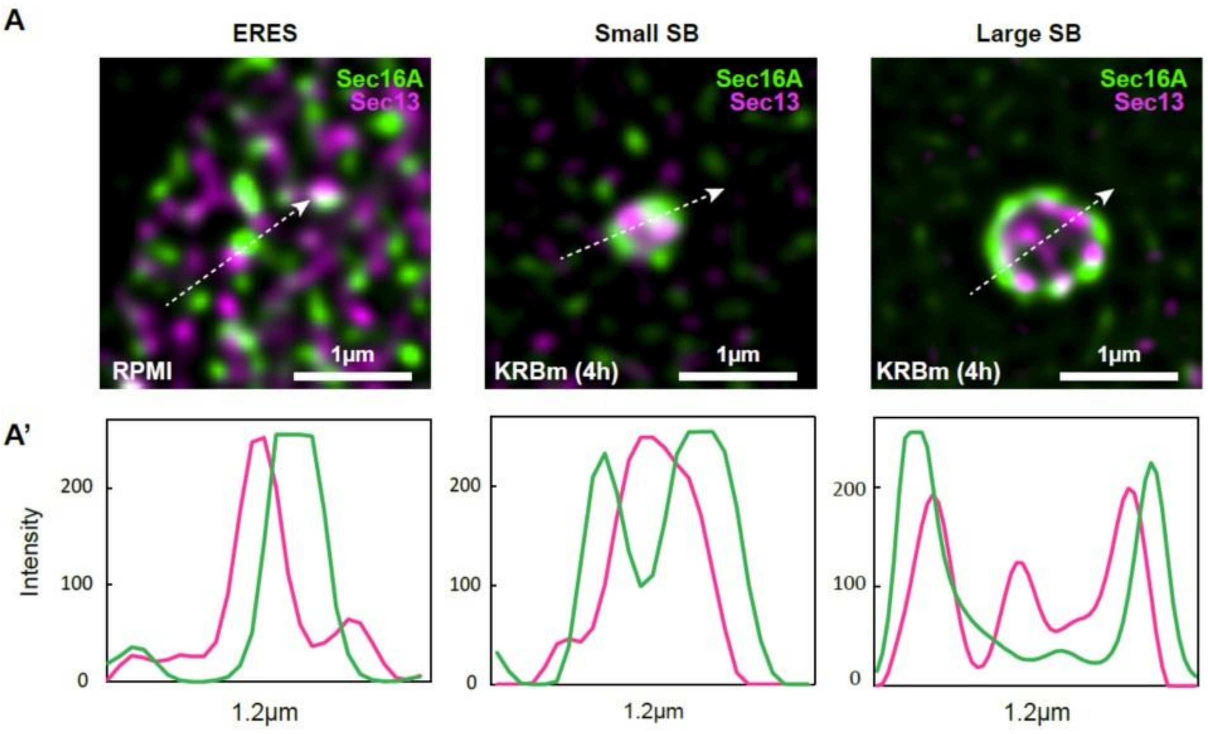

Figure 1 for Rev: Nanoscale organization of ERES proteins at ERES and in small and large Sec bodies. A: INS-1 cells in RPMI and KRBm for 4h were labeled for endogenous Sec16A (green) and Sec13 (magenta) that were visualized with STED microscope.
A': Intensity profile line.

© 2022. Published by The Company of Biologists under the terms of the Creative Commons Attribution License (https://creativecommons.org/licenses/by/4.0/).

Figure 2 for editor and reviewers.

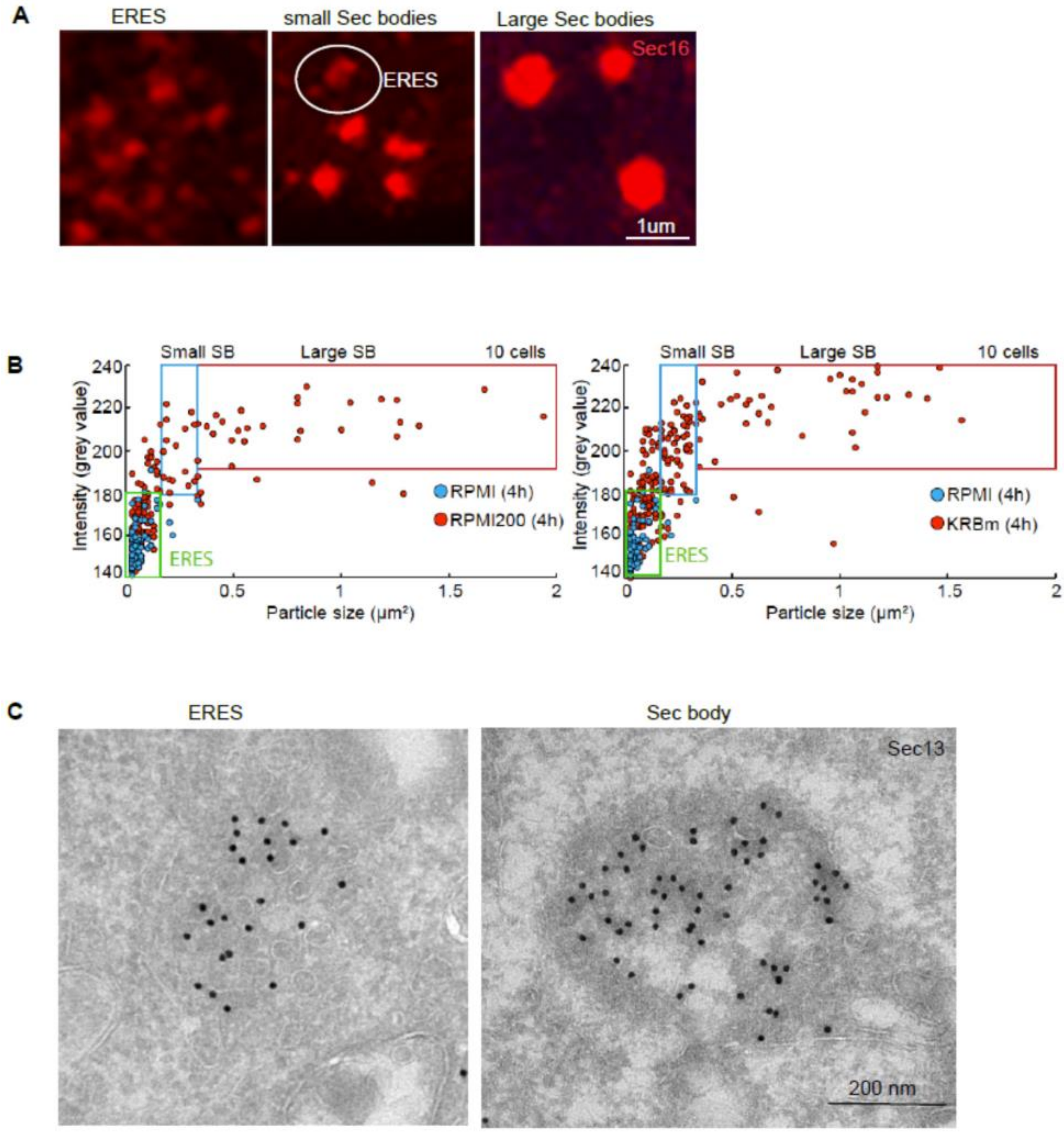

Figure 2 for Rev: Morphological and quantitative differences between ERES, small and large Sec bodies.

A: ERES (INS1cells in RPMI) and Sec bodies (INS1cells in KRB) by IF marking endogenous Sec16 (red). Note the clear difference in shape and intensity between ERES and Sec bodies. The white circle marks an ERES that has not been remodeled upon KRBm incubation.

B: Scatterplot depicting Sec16A foci size and intensity upon incubation in RPMI, RPMI200 and KRBm (4 h). ERES are contained in the green box; small sec bodies are contained in the blue box and the large sec bodies are contained in the red box, according to the definition described in the MM. The values within the red and blue box were used to determine the total intensity of Sec bodies (Sec body mean intensity * Sec body size) per cell and per condition. Foci from a total of 10 cells are displayed.

C: One ERES (INS1 cells in RPMI) and 1 Sec body (INS1 cells in KRN) viualised by immunogold labeling on frozen sections using an antibody to mark endogenous Sec13(15 nm gold).

© 2022. Published by The Company of Biologists under the terms of the Creative Commons Attribution License (https://creativecommons.org/licenses/by/4.0/).

Original submission

First decision letter

MS ID#: JOCES/2022/260294

MS TITLE: Stress-induced phase separation of ERES components into Sec bodies precedes ER exit inhibition in mammalian cells

AUTHORS: Wessel van Leeuwen, Dan TM Nguyen, Rianne Grond, Tineke Veenendaal, Ginny G Farias, and Catherine Rabouille
ARTICLE TYPE: Research Article

Thank you for submitting your work to us via Review Commons. We have now reached a decision on the above manuscript.

To see a copy of this decision letter, please go to: https://submit-jcs.biologists.org and click on the 'Manuscripts with Decisions' queue in the Author Area. (Corresponding author only has access to reviews.)

As you know, the reviewers raise a number of substantial criticisms that would prevent me from accepting the paper at this stage but I appreciate the clear and detailed revision plan that you have provided. I agree that this provides a sensible route to addressing the reviewers' concerns and that a revised version might prove acceptable.
Your revision plan already addresses what I agree to be the main issues - specifically the questions around defining ERES versus Sec bodies, the issues within Figure 7 highlighted by reviewer 1, some of the quantitative analysis, and the strength of conclusions that might be drawn. The BFA experiment is a sensible one given your previous data in Drosophila cells. You might also consider a more selective COPII inhibitor such as H89 or the Sar1-H79G mutant although of course these have their own caveats and issues. Overall, I agree with your revision plan and I would be pleased to see a revised manuscript which we would then return to the reviewers.

Please ensure that you clearly highlight all changes made in the revised manuscript. Please avoid using 'Tracked changes' in Word files as these are lost in PDF conversion.

I should be grateful if you would also provide a point-by-point response detailing how you have dealt with the points raised by the reviewers in the 'Response to Reviewers' box. Please attend to all of the reviewers' comments. If you do not agree with any of their criticisms or suggestions please explain clearly why this is so.

First revision

Author response to reviewers' comments

We thank the reviewers for their overall very positive comments and questions, and their support of the manuscript. Below, we have addressed all their points indicating the textual changes, clarifications and experimental approaches taken to revise the manuscript.

Reviewer #1 (Evidence, reproducibility and clarity)
Van Leeuwen et al. analyze and characterize Sec bodies in mammalian cells. The lab has discovered previously Sec bodies in Drosophila, which are membraneless organelles containing ER exit site (ERES) and COPII components and which form under specific stress conditions. They find that Sec bodies can also be formed at least in a rat cell line that highly secretes insulin, INS-1 under osmotic stress or a combination of amino acid starvation and osmotic stress. They establish that the ERES component Sec16A but not its isoform Sec16B is a driver of Sec body formation while still being a constituent of this organelle. Finally, the authors aimed to elucidate the relationship between Sec bodies and ERES and whether block ERES drives Sec body formation or whether Sec body formation causes a block in ER exit.

© 2022. Published by The Company of Biologists under the terms of the Creative Commons Attribution License (https://creativecommons.org/licenses/by/4.0/).

While most of the data provided is convincing, there are some shortcomings in particular with the RUSH experiments. The data on the regulation of ERES numbers by Sec bodies is not fully convincing. The authors could either tone down their conclusions or would need to provide more and stronger evidence.

Specific points
Major points
Rev1.1: The authors talk about remodeling of ERES into large Sec16A positive structures. It is unclear what the authors mean with remodeling. Sec16A is re-routed, changes its localization, but nothing more. There are no other ERES components are shown. Clearly, this is premature and an overinterpretation at this point to conclude that ERES goes into Sec16A positive structures, if the only marker that is shown in Sec16A. So, either the authors also show other ERES components (i.e combine Fig 1+2) or they need to re-write this part to stick with what they can conclude: Sec16A changes its localization under these specialized conditions.
>> We understand the concern of the reviewer that mentioning the stress-induced changes in Sec16A distribution as “ERES remodeling” in **Fig. 1** is premature without showing other ERES components. This comes in **Fig. 2** and **Fig. 3,** which are now mentioned earlier in the manuscript.

The reviewer suggests combining **Figs.1** and **2**. This could make the resulting figure crowded, especially with the incorporation of new data that we have obtained the nanoscale distribution of ERES proteins using STED. This shows that Sec bodies (small and large) contain both endogenous Sec16A and Sec13 (see revised **Fig. 2**).

Rev1.2: The number of independent experiments performed for various experiments is not clear. In some cases, where it is stated, it's only two experiments. This not up to the standard; at least three independent experiments should be provided.
>> We have added the information of number of independent experiments in all Figures.
In general, we have performed two independent experiments counting 30-50 cells for most of the experiments. For most of the data showed in the manuscript, the results are consistent between cells and between independent experiments, and with small variation for conditions tested.

Fig. 1 displays the quantification of representative experiments. Importantly this quantitative analysis has been collected from many experiments performed independently, and these results have been recapitulated in many subsequent experiments shown as controls used to compare other conditions, for instance reversion, and hexanediol (**Fig. 3**). These effects of these treatments are over 90%, and we have not deemed necessary to spend time and resources to perform a third experiment to confirm this significant effect.

About the Sec16A and B depletion (**Figs. 4A-B’**; **Fig. 5A-B’**), the question is not whether we can efficiently and reproducibly deplete these proteins. The question is whether their depletion has effect on Sec body formation, which we have monitored by analyzing many cells on 2 independent experiments for each Sec16A and Sec16B. There were hardly any variations between experiments and again, the effects were very large. When Sec16A is depleted to over 85%, Sec body formation is inhibited by 90%. When Sec16B is depleted to over 80%, Sec body formation is not inhibited. Furthermore, Sec16A depletion was monitored, cell by cell using immunostaining: The more depleted, the less Sec bodies formed.

For live cell imaging (**Fig. 6B**), we have quantified 11 transfected cells from 2 independent experiments. Data acquisition and analysis is extensive and very time consuming. We show individual cells in graph, with similar temporal kinetics for Sec body fusion events. In additional experiments, similar results were obtained, fast Sec body formation / fusion events with same Sec16A marker together with Sec16B (Figure 6C), or with Sec24D (**Fig. 6D**).

Overall, our experimental conditions have large effects or no-effects and without large variation between cells and independent experiments. Statistics differences are clear when comparing groups from at least two independent experiments. Furthermore, different markers and techniques have been used to show remodeling of ERES into Sec bodies. In this regard, we respectfully trust that repeating experiments for all the figures that that have been generated from only two independent experiments, is unnecessary. This would be a large amount of time and resources to confirm what we have significantly shown in the manuscript.

For the RUSH experiment (**Fig. 7**), we originally performed 4 experiments, two with Sec16A as a Sec body marker (quantification shown), and 2 with Sec13 (data not shown), leading to the same conclusion. We have now repeated the entire experiment using Sec16A as a marker for N=3 experiments. This has allowed us to better dissect the differences between the two groups of cells

© 2022. Published by The Company of Biologists under the terms of the Creative Commons Attribution License (https://creativecommons.org/licenses/by/4.0/).

for 3h KRB (those with ER exit activity versus those without). Detailed quantification is provided in revised **Fig. 7** and in new **Table S1**.

We have further detailed the Mander's coefficients, M1 and M2 for all markers used (Sec16A, Sec24 and Sec13) per cell, and per small and large SB (see revised **Fig. 2**).

Last, we have provided additional experiments to strengthen the correlation between Sec body formation and blockage in ER exit (see new *Figure S7*).

Rev1.3: The authors use the Mander's coefficient for co-localization analysis. They should give both directions as the COPII components might still be at the ER and allow some ER exit, even if the corralling protein Sec16A is reduced.
>> See also **Rev2.1**. We have now provided additional details on the Mander's coefficients (M1 and M2 values) for entire cells, as well as for both small and large Sec bodies (see revised **Fig.2**). Coefficients are very similar for large and small Sec bodies, showing that the incorporation of COPII components is concomitant to the coalescence of Sec16A.

Regarding active for ER exit, upon 4h KRB (containing Sec16 and COPII components), this is addressed in **Fig.7**. It is also possible that Sec16A would be recruited earlier and more efficiently than the COPII subunits that would remain at ERES and be functional for ER exit. However, this is not what was observed. There are remaining ERES containing Sec16A and COPII subunits but no ERES containing only COPII subunits.
Rev1.4: Sec16A is not quantitatively in Sec bodies. This is particularly obvious in Fig. 5C and D. Is the remainder of Sec16A still ER-associated and supports COPII vesicle budding?
Rev1.5: It is unclear why the incubation times vary from experiment to experiment (except for Fig. 6 and 7). They authors may want to provide a better rational why sometimes 1 hr, 2 hrs or 4 hrs are used
>> We agree with the reviewer that endogenous Sec16A is not quantitatively recruited in Sec bodies in **Fig. 5D**. This is because, in this experiment, the KRBm incubation has only been performed for 30min, a too short incubation to promote the robust formation of Sec bodies in wild type cells. In fact, it takes between 2 and 4h to induce a quantitative Sec body formation under this condition (see the time course now provided in *Figure S1 and* **Table 1**). The reason we have chosen such a short KRBm incubation is to assess whether overexpression of Sec16A (**Fig. 4**) or B (**Fig. 5**) accelerates the process of Sec body formation. A long incubation would diminish the dynamic range of the measurement (efficacy and kinetics). With using 30min, we can state that the Sec16A overexpression promotes Sec body formation (when compared to non-transfected cells), whereas this is not the case for Sec16B. These results are in line with the depletion approach showing that Sec16A, but not Sec16B, is a driver in Sec body formation. We have explained the reasoning of incubation time in the manuscript result section.

Rev1.6a: The issue with Figure 7 is manifold: The standard definition used by the authors for Sec bodies by size and fluorescence intensity is not useful in this instance. In using the RUSH system, the ERES will increase in size and hence also the Sec16 staining (compare Fig. 7A and Fig S6A).
>> As requested by the reviewer, we have now performed a systematic quantitative analysis comparing 4h DMEM + biotin (**Fig.7A**) and 4h DMEM, no biotin added (revised *Figure S6A*). The quantification is shown in *Figure S6D*. There is not any significant variation in average number, average size nor average intensity of Sec16A-positive structures when releasing cargo from the ER with biotin. We therefore respectfully disagree that cargo release significantly enlarges the ERES in this experimental setting, an enlargement that could have been confused with small Sec Bodies.

Rev1.6b: In Panel A. there are no Sec bodies counted, but they are similar in size to what they count as small Sec bodies. It seems as if they just arbitrarily decided what is an ERES and what is a Sec body. This is important because in panel 7B, most of the TfR has left the ER and there are many large Sec16A structures that might be small Sec bodies, but they are not counted as such. These data are very ambiguous.
>> The reviewer hints to the fact that we have not provided a clear distinction between an ERES and a small Sec body. In the revised manuscript, we now clearly provide our definition of ERES, small and large Sec bodies. ERES are usually fainter in endogenous Sec16 staining (or other COPII subunit) by immunofluorescence (IF) and have an irregular shape (not round) (see revised **Fig. 1A'**). When we analyze their number in cells grown in RPMI, we find that there are about 100 ERES per cell (**Fig. 7**, *Figure S6D*), a number similar to what is published in the field for other mammalian cells.

© 2022. Published by The Company of Biologists under the terms of the Creative Commons Attribution License (https://creativecommons.org/licenses/by/4.0/).

When using the quantification method we designed (**Fig. 1A''**), the ERES cluster to the bottom left corner of the graph (now marked by a green BOX in revised **Fig. 1A'')** with an average of 0.043 um^2 in size and an intensity below 70% of the maximal intensity particle (when comparing RPMI versus RPMI- 200 or KRBm, and imaged with same laser settings, see Materials and Methods).

By IEM, they are characterized, as they have been before in many cells, by the presence of an ER cup, "limiting" a collection of membrane, buds, tubules and vesicles marked by Sec13 (arrow in **Fig. 3A**)

Sec bodies are different structures that have not been arbitrary assigned. They are the structures visible in stressed INS1 cells with KRBm and RPMI200. The IF intensity of Sec16 (and all markers used) is higher than ERES, they are larger and tend to adopt a round shape (**Fig. 1A'**). Small Sec bodies have a size of 0.15-0.3um2 and an intensity > 75% of the max intense particle. Large Sec bodies have a size larger than 0.3um2 with an intensity above 80% of the max intense particle (**Fig. 1A', 1A''**). By IEM, they are very different from ERES, as they are slightly more electron dense. They do not contain membrane and vesicles, yet their Sec13 immunogold density appears equal or higher than at ERES. This is in total agreement with the increase intensity observed by IF.

Of note, not all Sec16 and Sec13 (or other COPII subunit) coalesce in Sec bodies upon stress. We still observe the presence of ERES upon stress (about 23 per cells) as shown in **Fig. 3A** (arrow), **Fig. 1A'**, (white circle), **Fig. 7C'** (graph) and *Table S1*.

Given these definitions and the careful observation and quantifications of images over many experiments, we can safely distinguish ERES from small Sec bodies by IF. We have used these definitions in all experiments, including **Fig. 7A**. We have provided this data in the revised manuscript to highlight the clear distinction between ERES and Sec bodies.

Last, we agree that expressing Sec body formation as 'total intensity Sec body per cell (that includes both small and large Sec body populations (intensity*size), is confusing. For a clearer interpretation of the data in **Fig.7**, we now display the total intensity of both small and large Sec body populations. The average number, average size and average intensity for all different structures (ERES, small and large SBs) are shown in *Table S1*.

We also present the results differently for a better comparison between the conditions. We have grouped the efficiency of ER exit by RUSH-TfR (**Fig. 7F**), the total intensity per cell (size*intensity) for small and large Sec bodies (**Fig. 7G**) and the numbers of ERES (**Fig. 7H**).

Rev1.6c: The author would need to use a ERES marker that does not go into Sec bodies, such as Sar1. This applies than also for the rest of the figure and makes it impossible to solidly conclude that Sec bodies regulate the number of ERES.
>> In order to determine whether ERES are still present, we appreciate the suggestion of analyzing an ERES component that does not go into Sec bodies. First, given the definition given above, ERES are still present after 4h stress (about ¼ of the RPMI condition).

We have used Sar1 as such a marker both endogenous and overexpressed as Sar1-GFP. In control conditions, Sar1-GFP is not strictly at ERES sites, but also along ER membrane, similar to what has been shown in previous studies (Yorimitsu & Sato, Mol Biol Cell, 2012; Kurokawa, et al., J Cell Sci., 2016). Endogenous Sar1 displays a similar pattern by antibody staining. It therefore makes it difficult to assess the number of remaining ERES (see **Figure for Reviewers**, [NOTE: We have removed a figure which was provided for the Reviewers in confidence.] page 10).

Furthermore, we have found that GFP-Sar1A and endogenous Sar1B re-locate from the ER into undefined structures that are close to, but not overlapping with, Sec bodies (see **Figure for Reviewers**, page 10). This is similar to what has been observed in *Drosophila* cells (Zachargianni et al, 2014). This Sar1 behavior in stress appears to uncover a different biological process altogether that would require much more work, outside the scope of this present study.

Taken together, given that Sec16A/B, Sec23, Sec24 and Sec13 are present in INS-1 Sec bodies (as shown in different Figures of our manuscript), recruited in a concomitant manner as Sec16A, we propose that without any of these components, essential for COPII coat formation that are recruited to Sec bodies, less ERES are functional in ER export resulting in its inhibition.

Rev1.6d: Also trying to correlate number of ERES/cell with Total intensity of Sec bodies/cell is not something easily digestible. A fair comparison would be the 'relocalization'/changes in fluorescence intensities from ERES to Sec bodies, but for this a clear distinction of Sec bodies and ERES would be needed. A possibility would be that the authors take out Figure 7 altogether and refrain from making this conclusion.

© 2022. Published by The Company of Biologists under the terms of the Creative Commons Attribution License (https://creativecommons.org/licenses/by/4.0/).

>> We agree that **Fig. 7**, where we have not separated small and large Sec bodies, is confusing. For a clearer interpretation of the data, we have changed 'total intensity Sec body per cell' graph to display the intensities of both small and large Sec body populations, separately. In addition, we have incorporated a new *Table S1* showing changes in average number, size and intensity for ERES, small and large Sec bodies in 0, 1h, 3h (ER exit activity), 3h (no ER exit activity) and 4h KRBm. Differences between 0h and 4h KRB is also well represented in **Fig. 1A''**.

Here, we take the opportunity to stress that what critically controls secretion is NOT the amount of Sec bodies formed (large and small), but how much ERES are left to support ER exit. We propose that a certain threshold of ERES number (above a quarter) is needed to support proper secretion measured by RUSH. Of note, it is correct that these remaining ERES could be altered in other ways, thus not supporting ER exit for reasons others than their progressive depletion of Sec16 and COPII subunits. We have discussed this in the revised manuscript.

Minor points:
- The summary of the results at the end of the introduction is almost as long as the entire introduction and too many details are given. The introduction should be streamlined.
>> We have now shortened the summary at the end of the introduction as requested.

- P. 4, 3rd paragraph: The authors did not test a large set of Golgi proteins, but rather a set of large Golgi proteins.
>> We have rephrased this.

- P.7, 2nd last paragraph. Why should TfR ever get in a membraneless compartment? TfR contains a transmembrane domain and is normally an obligate dimer (i.e. 2 TMDs/complex). This statement does not make sense and should be removed.
>> Indeed, Sec bodies are not sealed by a membrane (**Fig. 3**). As they derive from ERES components and form where ERES were present, they could potentially have encapsulated several COPII coated membrane derived from the ER and therefore containing cargo.

Significance:
The authors provide convincing evidence for Sec bodies also at least in one mammalian cell line.

Therefore, their data imply that the larger dataset previously published by the group using Drosophila S2 cells might be generalisable for some mammalian cell lines with respect to osmotic stress response (+ aa starvation). They nicely show different roles for the ERES scaffold proteins Sec16A and Sec16B in the process of Sec body formation. The paper is a nice addition to the still relative scarce literature on secretion/membrane traffic under stress.
>> Thank you. We also want to point that we have established Sec body formation in other mammalian cells, i.e., primary rat neurons. This data has now been added to this manuscript (revised *Figure S2*). Sec body formation upon stress is therefore generalizable to more than one mammalian cell type.

Reviewer #2 (Evidence, reproducibility and clarity):
This study examines the formation of a stress-responsive membraneless compartment in mammalian cells that regulates secretion. Building on previous work in Drosophila S2 cells, which identified SEC bodies that sequester COPII trafficking machinery in response to nutrient and salt stress, the current work surveys mammalian cells for similar effects, finding that INS-1 cells also activate this pathway. Through a series of elegant, well-performed and thorough imaging analyses, the authors convincingly show that the mammalian system closely mirrors that of Drosophila. The findings are clearly presented, convincing and appropriately interpreted.
I have several relatively minor comments that might improve the manuscript for the reader.

Rev2.1. Figure 2: do small SEC bodies have less co-localization with COPII proteins than larger ones? This might suggest progression from an early, small Sec16-positive structure to larger COPII-positive structures. I was also curious as to whether there was a difference in co-localization intensity (Mander coefficient) between inner and outer coat components in early versus late (i.e. small versus large) SEC bodies.
>> We appreciate the request for clarification from the reviewer. In the original **Fig. 2**, we had shown the overlap of two endogenous proteins per cell. To distinguish the marker's overlap in small

© 2022. Published by The Company of Biologists under the terms of the Creative Commons Attribution License (https://creativecommons.org/licenses/by/4.0/).

and large Sec bodies, we have now quantified this overlap per structure (for small and large Sec bodies, separately) by using ROI Mander's (see revised **Fig. 2C**).

In addition, we have got a better understanding of the distribution of these proteins at the nanoscale level by STED super-resolution microscopy for endogenous Sec16A and Sec13, showing that in both small and large Sec bodies, Sec16A forms a "shell" around Sec13 (see revised **Fig. 2D**).

Last, we would like to point to the reviewer's attention that we have performed (and incorporated) dual live cell imaging using tagged Sec16A/Sec16B, Sec16A/Sec24D (**Fig. 5E, 6C-D** and *Figure S5*) showing in kinetic detail the concomitant co-distribution of ERES proteins during the remodeling from ERES to small and large structures. The coalescence of the ERES markers occur at the same time as Sec16A.

Rev2.2. Figure 3A: in the left panel, the right-hand cluster of Sec13 labelling seems to be associated with membrane structures. Is this remnant ERES?
>> The reviewer is correct. The mentioned structure it is an ERES that have not been remodeled. It has the same feature as the ERES in cells grown in RPMI. This is now mentioned in a clearer manner in the Figure and Figure legend.

Rev2.3a: Figure 7 D/E: I found the initial description of these experiments confusing. The authors should state clearly that D and E represent the two different populations of cells under the same incubation conditions.

>> We apologize as we thought the text was clear in this regard. The two categories of cells (incubated for 3h in KRBm) are made by sorting the cells that display ER exit activity from those that do not (using RUSH).

In the cells where ER exit can be visualized (TfR out of the ER), the number of ERES is around 54. In the cells where no ER exit can be visualized (TfR in the ER), the number of ERES is 27. This number is similar to cells incubated for 4h in KRBm where no ER exit is visualized (23 ERES). We have hopefully explained this better in the manuscript. For clarification, we have also changed the graphs to compare different groups of cells (**Fig. 7F-H**), including both populations of cells (ER exit and no ER-exit) for 3h KRBm.

Rev2.3b: It was also unclear to me how the conclusion was reached from this experiment that SEC body formation preceded ER exit inhibition. To my mind, there is simply a correlation between SEC body size/extent and secretion defects. This should be spelled out more clearly.
>> The reviewer is correct that the correlation between Sec body formation and ER exit inhibition is misleading. In fact, what we propose is that there is a correlation between ER exit inhibition and the number of functional ERES left and still competent for transport. We propose that Sec body formation titrates away Sec16 and COPII subunits away from ERES, making them unfunctional.

What we say is that Sec body formation precedes the inhibition of ER exit and is therefore not a consequence of ER inhibition. To show this more firmly, we have performed an experiment with BFA that blocks traffic in the early secretory pathway and ultimately blocks ER exit in 99% of the cells. As in Drosophila cells (Zacharogianni et al, 2014), we now clearly show that BFA incubation does not lead to Sec body formation (*Figure S7A, A'*).

Upon request from the editor, we have also used H89 (that leads to the translocation of Sar1 away from the ER membrane and causes ER exit inhibition). Upon H89 treatment described in the manuscript, around 53% of the cells display inhibition of ER exit. Similarly, Sec bodies do not form in these cells and the ERES number is similar to control cells (both untreated and those treated but where ER exit still occurs) (*Figure S7B, B'*). Taken together, we conclude that inhibiting ER exit does not lead to Sec body formation.

It is correct that we have mentioned that Sec body formation causes ER exit inhibition by titrating away key components. However, we acknowledge that there might be other causes to this inhibition, such as modifications and other effects of the stress to other part of the machinery that are independent of the ERES remodeling we report here. This has been added to the discussion section.

Rev2.3c: Similarly, the method for defining what corresponds to an ERES versus a small SEC body should be more clearly stated in the results here so that the reader can understand the distinction and subsequent quantification.

© 2022. Published by The Company of Biologists under the terms of the Creative Commons Attribution License (https://creativecommons.org/licenses/by/4.0/).

>> The reviewer hints to the fact that we have not provided a clear distinction between an ERES and a small Sec body. In revised manuscript, we now clearly provide our definition of ERES, small and large Sec bodies. ERES are usually fainter in endogenous Sec16 staining (or other COPII subunit) by immunofluorescence (IF) and have an irregular shape (not round) (see revised **Fig. 1A'**). When we analyze their number in cells grown in RPMI, we find that there are about 100 ERES per cell (**Fig. 7**, *Figure S6D*), a number similar to what is published in the field for other mammalian cells.

When using the quantification method we designed (**Fig. 1A''**), the ERES cluster to the bottom left corner of the graph (now marked by a green BOX in revised **Fig. 1A'')** with an average of 0.043 um^2 in size and an intensity below 70% of the maximal intensity particle (when comparing RPMI versus RPMI- 200 or KRBm, and imaged with same laser settings, see Materials and Methods).

By IEM, they are characterized, as they have been before in many cells, by the presence of an ER cup, "limiting" a collection of membrane, buds, tubules and vesicles marked by Sec13 (arrow in **Fig. 3A**)

Sec bodies are different structures that have not been arbitrary assigned. They are the structures visible in stressed INS1 cells with KRBm and RPMI200. The IF intensity of Sec16 (and all markers used) is higher than ERES, they are larger and tend to adopt a round shape (**Fig. 1A'**). Small Sec bodies have a size of 0.15-0.3um2 and an intensity > 75% of the max intense particle. Large Sec bodies have a size larger than 0.3um2 with an intensity above 80% of the max intense particle (**Fig. 1A', 1A''**). By IEM, they are very different from ERES, as they are slightly more electron dense. They do not contain membrane and vesicles, yet their Sec13 immunogold density appears equal or higher than at ERES. This is in total agreement with the increase intensity observed by IF.

Of note, not all Sec16 and Sec13 (or other COPII subunit) coalesce in Sec bodies upon stress. We still observe the presence of ERES upon stress (about 23 per cells) as shown in **Fig. 3A** (arrow), **Fig. 1A'**, (white circle), **Fig. 7C'** (graph) and *Table S1*.

Significance:
This study represents a significant advance in that it reveals a conserved stress-response pathway that acts in professional secretory cells. There is little new mechanistic insight (eg. the singling pathways or PTMs that contribute to SEC body formation) but that does not detract from the significance of the findings. The work will be of interest to researchers in the protein trafficking field, as well as those interested more broadly in liquid-liquid phase separation and membraneless organelles.
>> Thank you.

Reviewer #3 (Evidence, reproducibility and clarity):
This group previously identified Sec bodies as membraneless organelles that form in Drosophila S2 cells upon NaCl stress or amino acid starvation to encapsulate components of ER exit sites (ERES). Here they demonstrate that Sec bodies also form under similar stress conditions in highly secretory mammalian INS-1 cells. A key component of mammalian Sec bodies is Sec16A, while the smaller Sec16B protein is a component of Sec bodies but is not a driver of their formation. The results indicate that sequestration of Sec16A inhibits ER exit by reducing the number of ERES below a critical threshold. This inhibition is readily reversible when the stress is relieved. The conclusion is that Sec16A-driven formation and dissolution of Sec bodies is a mechanism to regulate ER exit in response to environmental conditions.

Rev3.1: I have only one minor issue with the data. Knockdown of Sec16A inhibits Sec body formation, but presumably ER exit will also be blocked. If so, how can indirect effects of the Sec16A knockdown be ruled out? This result is suggestive of a crucial role for Sec16A in Sec body formation but is not definitive, and the interpretation should be appropriately cautious.

>> We appreciate the question from the reviewer. Indeed, Sec16A depletion severely reduce secretion (Bhattacharyya & Glick, 2007) and in this regard, it is possible that this ER exit inhibition is the cause of Sec body formation. However, there are two arguments against this: First, upon Sec16A depletion that severely reduces secretion and ER exit (Bhattacharyya & Glick, 2007), did not lead to Sec body formation in cells maintained in RPMI (**Fig. 4B'**). Second, Sec16B depletion also leads to ER exit inhibition (Bhattacharyya &Glick, 2007), but Sec bodies still form upon Sec16B depletion upon KRBm treatment, whereas Sec16A depletion prevents Sec body formation (**Fig. 4B**, **Fig. 5B**).

© 2022. Published by The Company of Biologists under the terms of the Creative Commons Attribution License (https://creativecommons.org/licenses/by/4.0/).

We have now incorporated additional experiments with BFA and H89 to assess the role of ER exit inhibition in Sec body formation. As in Drosophila, INS-1 cell incubation with BFA (in RPMI) does not induce Sec body formation (*Figure S7A, A'*). Nor does H89 incubation (*Figure S7B, B'*). We conclude that a block in ER exit (and trafficking in the early secretory pathway) does not lead to Sec body formation.

To test whether secretion during KRBm treatment would have a role to play in Sec body formation, we incubated cells in KRBm with BFA. This did not alter Sec body formation compared to KRBm alone (*Figure S7C, C'*).

Significance:

The main takeaway message is that the Sec body formation initially discovered in invertebrates also occurs in at least some mammalian cells. As such, the novelty of the study is only moderate. On the other hand, the finding that Sec body formation is a widespread phenomenon is a significant advance. Moreover, this work extends the prior data from Drosophila with new insights obtained by characterizing the composition, dynamics, and functions of mammalian Sec bodies. The experiments are exceptionally clean and elegant, and they are presented well. For these reasons, the paper will be of broad interest to researchers interested in membrane traffic and membraneless organelles.

>> Thank you.

Second decision letter

MS ID#: JOCES/2022/260294

MS TITLE: Stress-induced phase separation of ERES components into Sec bodies precedes ER exit inhibition in mammalian cells

AUTHORS: Wessel van Leeuwen, Dan TM Nguyen, Rianne Grond, Tineke Veenendaal, Catherine Rabouille, and Ginny G Farias
ARTICLE TYPE: Research Article

We have now reached a decision on the above manuscript.

To see the reviewers' reports and a copy of this decision letter, please go to: https://submit-jcs.biologists.org and click on the 'Manuscripts with Decisions' queue in the Author Area. (Corresponding author only has access to reviews.)

As you will see, the reviewers gave favourable reports but one raised two minor points that I consider would nicely polish your manuscript. I hope that you will be able to carry these out because I would like to be able to accept your paper, depending on further comments from reviewers. I would not then need to return it to the reviewers.

Please ensure that you clearly highlight all changes made in the revised manuscript. Please avoid using 'Tracked changes' in Word files as these are lost in PDF conversion.

I should be grateful if you would also provide a point-by-point response detailing how you have dealt with the points raised by the reviewers in the 'Response to Reviewers' box. Please attend to all of the reviewers' comments. If you do not agree with any of their criticisms or suggestions please explain clearly why this is so.

Reviewer 1

Advance summary and potential significance to field

This revised manuscript satisfactorily addresses my previous (minor) concerns when I reviewed for Review Commons.

© 2022. Published by The Company of Biologists under the terms of the Creative Commons Attribution License (https://creativecommons.org/licenses/by/4.0/).

Comments for the author

I have a couple of editorial suggestions that stem from other reviewers' comments. These suggestions are only suggestions, but I think would clarify the paper nicely.

1. Regarding the term "remodelled" - I agree with the other reviewer that this term is perhaps not appropriately used with regard to SEC bodies and protein localisation. Generally, the term is used to describe morphological changes to a structure: membranes, or a large protein complex. ERES might be remodelled but SEC16 itself is rather redistributed, as are the COPII proteins. Remodelled implies it is the same structure but a different shape. Here, however, the SEC body is a completely new structure unrelated to ERES (being free of membranes).

2. Regarding the use of 2 biological replicates with multiple cells sampled in each experiment. I suggest that datapoints from the two independent experiments are represented with distinct colours so that the reader can clearly see that there is little variability between experiments. This is becoming standard now in the field ("SuperPlots").

Reviewer 2

Advance summary and potential significance to field

Endoplasmic reticulum exit sites (ERES) are sites where proteins are packaged into COPII vesicles destined to the Golgi apparatus, thus the entrance into the secretory pathway. The authors show that under stress ERES are remodelled with components being deposited into SEC bodies, and thereby shutting down ER ext.

Comments for the author

The authors have addressed my concerns and I recommend publication of this nice paper.

Second revision

Author response to reviewers' comments

We thank the reviewers for their overall very positive comments and their support of the manuscript. Below, we have addressed minor points indicating the textual changes and clarifications for reviewer 1.

Reviewer 1 Advance summary and potential significance to field
This revised manuscript satisfactorily addresses my previous (minor) concerns when I reviewed for Review Commons.

Reviewer 1 Comments for the author
I have a couple of editorial suggestions that stem from other reviewers' comments. These suggestions are only suggestions, but I think would clarify the paper nicely.
1. Regarding the term "remodelled" - I agree with the other reviewer that this term is perhaps not appropriately used with regard to SEC bodies and protein localisation. Generally, the term is used to describe morphological changes to a structure: membranes, or a large protein complex. ERES might be remodelled but SEC16 itself is rather redistributed, as are the COPII proteins. Remodelled implies it is the same structure but a different shape. Here, however, the SEC body is a completely new structure unrelated to ERES (being free of membranes).
R: We have now replaced the term 'remodeled' with redistributed/reorganized/coalesced.

2. Regarding the use of 2 biological replicates with multiple cells sampled in each experiment. I suggest that datapoints from the two independent experiments are represented with distinct

© 2022. Published by The Company of Biologists under the terms of the Creative Commons Attribution License (https://creativecommons.org/licenses/by/4.0/).

colours so that the reader can clearly see that there is little variability between experiments. This is becoming standard now in the field ("SuperPlots").
R: We have included data-points in our graphs, with distinct colors for independent experiments.

Reviewer 2 Advance summary and potential significance to field
Endoplasmic reticulum exit sites (ERES) are sites where proteins are packaged into COPII vesicles destined to the Golgi apparatus, thus the entrance into the secretory pathway. The authors show that under stress ERES are remodelled with components being deposited into SEC bodies, and thereby shutting down ER ext.

Reviewer 2 Comments for the author
The authors have addressed my concerns and I recommend publication of this nice paper.
R: Thanks

Third decision letter

MS ID#: JOCES/2022/260294

MS TITLE: Stress-induced phase separation of ERES components into Sec bodies precedes ER exit inhibition in mammalian cells

AUTHORS: Wessel van Leeuwen, Dan TM Nguyen, Rianne Grond, Tineke Veenendaal, Catherine Rabouille, and Ginny G Farias
ARTICLE TYPE: Research Article

Thank you for sending your manuscript to Journal of Cell Science through Review Commons.

I am happy to tell you that your manuscript has been accepted for publication in Journal of Cell Science, pending standard ethics checks. Thank you for submitting your work to us.

© 2022. Published by The Company of Biologists under the terms of the Creative Commons Attribution License (https://creativecommons.org/licenses/by/4.0/).
